# Multi‐Tissue Crosstalk Regulating Meniscus Healing Under Inflammation and Physiological Loading

**DOI:** 10.1002/advs.77758

**Published:** 2026-09-16

**Authors:** Hun Jin Jeong, Elen Zhu, Min Seo Kang, Ming Wang, Sam Comito, Chang H. Lee

**Affiliations:** ^1^ Biomedical Engineering University of Maryland East Shore Princess Anne Maryland USA; ^2^ College of Dental Medicine Columbia University Irving Medical Center New York New York USA

**Keywords:** avascular meniscus healing, bioactive glue, bioreactor, mechanical stimulation, meniscus‐on‐a‐chip, multi‐tissue crosstalk in knee joint

## Abstract

The meniscus has been largely ignored in the emerging research interest in multi‐tissue crosstalk in knee joints. This study discovers the novel roles of three‐way crosstalk between meniscus, synovial membrane, and fat in the regulation of meniscus injury, degeneration, and healing. A novel meniscus‐on‐a‐chip (MoC) model is developed, equipped with a bioprinted synovial membrane, engineered fat tissue, and a meniscus explant healing model, treated with bioactive glue and synovial mesenchymal stem cells (syMSCs). Our MoC integrated with a meniscus‐specific bioreactor enables a comprehensive investigation into the combinational effects of inflammation and physiological/pathological loading on meniscus healing and degeneration. The MoC successfully replicates not only meniscus healing induced by bioactive glue but also the OA initiation and progression from meniscus injury. Comprehensive scRNA‐seq with CellChat analysis suggests how communications between adipocytes, macrophages, fibrochondrocytes, and syMSCs regulate the fate of meniscus injury in response to exogenous stimulation. This study represents the first identification of cell‐cell communication signals between meniscus, synovial membrane, fat, and syMSCs, which are potentially responsible for regulating meniscus healing under inflammation and mechanical loading. These novel findings may have significant implications for developing next‐generation therapeutics for meniscus injury and prevention of joint degeneration through regulating multi‐tissue crosstalk in the joint.

## Graphical abstract

Using a novel Meniscus‐on‐a‐chip (MoC) model integrated with a meniscus‐specific bioreactor, this study investigated the roles of multi‐tissue crosstalk that regulates injury, healing, and degeneration of the meniscus injury under inflammation and pathophysiological loading. Comprehensive scRNA‐seq with CellChat analysis suggests how communications between adipocytes, macrophages, fibrochondrocytes, and syMSCs regulate the fate of meniscus injury in response to exogenous stimulation. This study represents the first identification of cell‐cell communication signals between meniscus, synovial membrane, fat, and syMSCs, which are potentially responsible for regulating meniscus healing under inflammation and mechanical loading. These novel findings may have significant implications for developing next‐generation therapeutics for meniscus injury and prevention of joint degeneration through regulating multi‐tissue crosstalk in the joint.

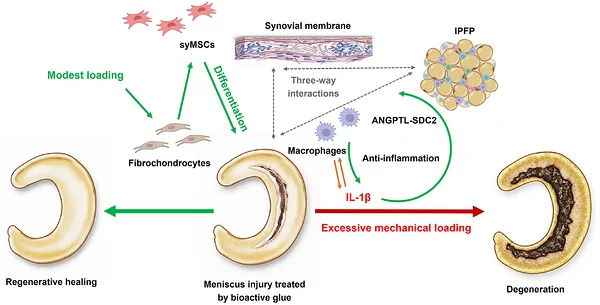

## 1 Introduction

Knee menisci are crescent‐shaped fibrocartilaginous tissues, playing essential roles in load transmission, shock absorption, and joint stability. Tears in the vascularized outer third region of the meniscus can be surgically repaired by suturing torn parts. In contrast, tears in the inner avascular region, similar to articular cartilage, cannot be repaired due to poor intrinsic healing capacity and frequently extend into the middle‐third region, followed by meniscus deterioration [1, 2, 3, 4]. Even with repairs, meniscus tears in both the vascularized and avascular zones significantly increase the risk of osteoarthritis (OA) [5]. Consequently, meniscal tears remain one of the most common orthopedic injuries, frequently necessitating partial or total meniscectomy. While meniscectomy provides short‐term symptomatic relief, it fundamentally alters joint contact mechanics, significantly increasing the risk of secondary OA and long‐term joint degeneration [6]. Each year, over one million patients undergo surgical meniscus repair or meniscectomy in the U.S. [3, 7]. Despite the prevalence, quality of life impact, and health care burden of meniscus injuries, there is no clinically applicable treatment to induce functional healing of avascular meniscus tears reliably.

To address the clinical needs, tissue engineering and regenerative medicine have emerged as promising alternatives for restoring functional tissue by using bioactive scaffolds, growth factors, and stem cells [8, 9, 10, 11, 12, 13, 14]. For instance, intra‐articular injections of synovium‐derived mesenchymal stem cells (syMSCs) have demonstrated potential to promote meniscal regeneration in animal models and human patients [15, 16, 17]. Injectable bioadhesives/bioglue hydrogel systems have been explored to mechanically seal tears and deliver therapeutic agents, offering a minimally invasive solution to stabilize defects [8, 9]. Our group has recently reported bioactive glues designed to enhance regenerative healing in avascular tears by activating endogenous stem/progenitor cells [10, 11, 12]. However, clinical translation of bioengineering approaches for meniscus healing remains challenging despite promising results in isolated environments. One of the primary limitations of current tissue engineering strategies is that the designed functionality has been predominantly focused on treating the meniscus in isolation. However, the existing in vitro models for meniscus injury and healing mostly fail to recapitulate the complex, multi‐tissue microenvironment of the knee joint, and the existing scaffolds do not aim to regulate the multi‐tissue crosstalk [18].

A knee joint is a complex multi‐tissue organ encompassing the articular cartilage, menisci, ligaments, fat, and synovial membrane. Given the crucial roles of the multi‐tissue crosstalk between the joint components in homeostasis and disease initiation and progression, animal models have been widely used to test investigational therapies for injured or degenerated joint tissues [19, 20]. However, animal models have limitations such as differences from human joint conditions, insufficient reproducibility, longer timelines, higher costs, and ethical issues [19, 20]. Accordingly, there is an emerging research interest in the efficient and reliable Joint‐on‐a‐chip (JoC) model to study new drugs, disease mechanisms, and regenerative therapies [19, 20]. The JoC models possess notable advantages, including more reproducible data, controlled experimental parameters, and a lower cost [19, 20]. However, the existing in vitro knee joint models have been limited to synovium‐on‐a‐chip, cartilage/osteochondron‐on‐chip, or a miniature joint focusing on OA [19, 20, 21]. OA patient‐derived explant co‐culture models were also utilized to study interactions between synovium and cartilage/osteochondral tissue [22, 23], which are, however, limited by the scarce availability of appropriate human tissues with consistent disease status. Notably, the meniscus has been missing in all existing JoC and explant co‐culture models, resulting in our limited understanding of the roles of multi‐tissue crosstalk in meniscus injury, healing, and degeneration [19, 24, 25].

In this study, we developed a novel meniscus‐on‐a‐chip (MoC) model to investigate the roles of crosstalk between the meniscus, the synovium, and the infrapatellar fat pad (IPFP) in meniscus injury and healing. The synovium and IPFP were selected for the MoC given their critical roles in joint diseases and homeostasis, closely associated with meniscus injury, healing, and degeneration [26, 27, 28, 29, 30]. The synovium serves as a reservoir for syMSCs and macrophages, which orchestrate the inflammatory cascade following meniscus injury [27, 28]. Macrophage polarization, shifting from a pro‐inflammatory M1‐like phenotype to a regenerative M2‐like phenotype, is perceived as a critical determinant of joint tissue integration and healing outcomes [27, 28]. Simultaneously, the IPFP is a metabolically active depot of adipocytes that secretes a variety of adipokines and cytokines capable of modulating inflammation and cartilage health [29, 30]. Although the functions of synovium and IPFP have been investigated in the scope of OA initiation and progression [26, 27, 29, 30], their roles have rarely been explored for meniscus injury and healing. Using a validated MoC model, comprising the meniscus, synovium, and IPFP, our study discovered the critical roles of three‐way crosstalk between the tissues in the regulation of meniscus healing under inflammation.

In addition, our study elucidates the underexplored roles of multi‐tissue crosstalk, not only under inflammation but also under physiological loading. Although physiological loading contributes to meniscus health and healing [31], pathophysiological loading derived from an injury‐induced mechanical environment can be detrimental, leading to meniscus degeneration [32, 33]. Previous studies suggested that pathophysiological loading affects upregulated pro‐inflammatory cytokines [34]. In contrast, a controlled laboratory study with a meniscus explant healing model showed that physiological loading can attenuate the detrimental effects of IL‐1β on meniscus healing [35]. Despite the research progress, there is still a big knowledge gap between the mechanical loading‐inflammation interplay and the degenerative progression of meniscus injuries [34]. To address the gap, we integrated the MoC with a meniscus‐specific bioreactor, allowing the application of physiological loading in combination with pro‐inflammatory cytokines. Our study with the novel MoC integrated with a bioreactor provides new insights into the pathophysiology of meniscus injury that can serve as a scientific foundation for developing potential therapeutic targets for enhancing meniscus regeneration in a complex joint environment.

## 2 Results

### 2.1 Design and Fabrication of MoC

**FIGURE 1:**
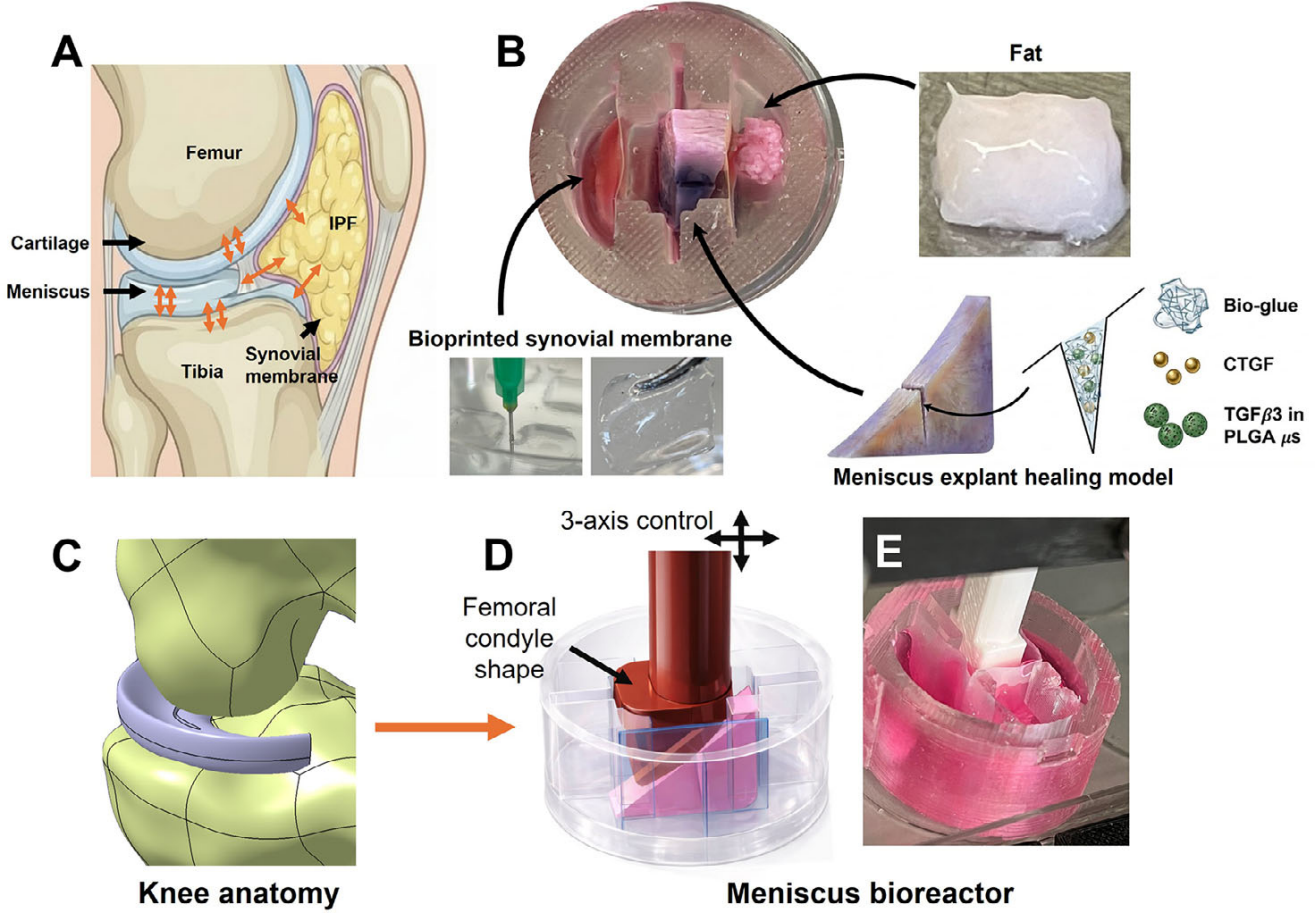
Construction of a Meniscus‐on‐a‐Chip (MoC) model. To recapitulate the crosstalk between joint tissues in the knee joint (A), multi‐chamber compartments were built using PDMS with semi‐permeable membranes, allowing co‐culture of a meniscus explant undergoing injury healing with bioactive glue with bioprinted synovial membrane and engineered fat tissue (B). Based on human knee anatomy (C), mechanical loading units were created with the shape of the femoral condyle (D). 3D‐printed loading units were equipped with MoC and a bioreactor with 3‐axes motion controlling unit (E).

To recapitulate the multi‐tissue environment in the knee joint (Figure 1A), the meniscus‐on‐a‐chip (MoC) was designed to have multiple tissue compartments, including a joint cavity for the knee meniscus, synovium, and fat (Figure 1B). The synovial membrane was bioprinted as a bilayer of syMSCs and macrophages, as described below, and fat was engineered using a 3D‐printed hybrid scaffold infused with collagen gel and adipose tissue‐derived stem cells (ADSCs) (Figure 1B). The primary tissue culture chamber was designed to secure a wedge‐shaped meniscus explant where a longitudinal incision will be made, followed by treatment with bioactive glue, sequentially releasing connective tissue growth factor (CTGF) and transforming growth factor beta 3 (TGF‐β3) from polymeric microspheres (µS), per our prior methods [10, 11, 12] (Figure 1B). The chamber was fabricated using 3D‐printed molds with polydimethylsiloxane (PDMS). The primary tissue chamber (Figure 1B) was incorporated and partitioned by a semi‐permeable membrane to facilitate discrete tissue compartments while allowing controlled nutrient and fluid exchange between sections. The chip was designed using a 3D CAD at dimensions of 34 mm x 17 mm (dia. x height) and fabricated with PDMS using a digital light processing (DLP)‐printed mold (Figure S1). From a pilot permeability test with 70 kDa dextran dyes, we selected a semi‐permeable membrane with 20 µm pores (Figure S2). Based on the anatomical structure of a human knee joint (Figure 1C), the condylar‐shaped mechanical loading unit was created (Figure 1D). The loading unit was constructed using 3D‐printed biocompatible poly(lactic acid) (PLA) that was equipped with 3‐axis motion control units with a stroke range of 150‐mm the x‐, y‐, and z‐axes and a 100 µm resolution in the location control, as described in our previous work [36] (Figure 1E). The bioreactor system consisted of stimulation components, a well‐plate holder, and a heating bed to maintain temperature (Figure 1E).

### 2.2 Bioprinting Synovial Membrane

**FIGURE 2:**
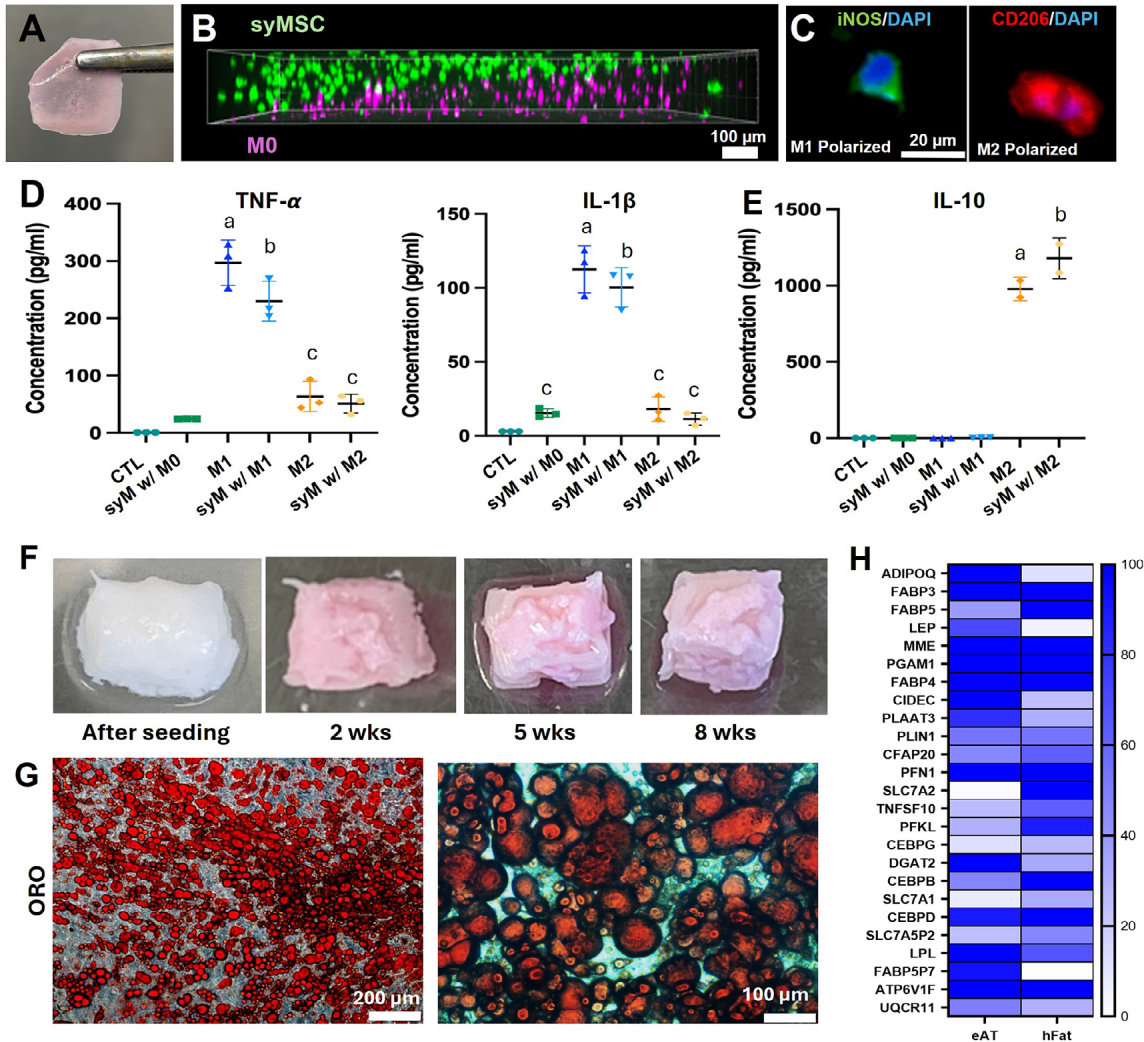
Engineered synovial membrane (syM) and fat. The syM was bioprinted as a bilayer of syMSCs and THP‐1‐derived macrophages in GelMA (A, B). Macrophages in the bioprinted syM were polarized into iNOS^+^ M1‐like and CD206^+^ M2‐like macrophages upon LPS/IFN‐γ and IL‐4/IL‐13 stimulation, respectively (C). Pro‐inflammatory cytokines, TNF‐α and IL‐1β, were significantly increased in monolayer macrophages polarized to M1 and syM with M1 stimulation (D) (n = 3 per group; p<0.001; different letters indicate significant differences). Similarly, anti‐inflammatory IL‐10 was significantly increased in monolayer macrophages polarized to M2 and syM with M2 stimulation (E) (n = 3 per group; p<0.001; different letters indicate significant differences). By 8 weeks of culture in adipose differentiation media, a mature adipose tissue was engineered in an ADSC‐seeded collagen/PCL‐hybrid scaffold (F). Oil Red O (ORO) staining shows abundant lipid droplets formed in the engineered fat tissue by 8 weeks (G). RNA‐seq shows that genes enriched in human joint fat tissue were expressed in the engineered fat tissue (H).

Synovial membrane (syM) was constructed using a multi‐channel bioprinter, as human Tohoku Hospital Pediatrics‐1 (THP‐1) cell‐derived macrophages were printed via our gelatin‐based bioink [37], then printing P2 – 3 human synovial MSCs on top of the macrophage lining (Figure 2A,B). The macrophages were derived from THP‐1 human monocytes by culturing in complete RPMI media (ThermoFisher Scientific, Waltham, MA), supplemented with 10% heat‐inactivated FBS. To differentiate THP‐1 monocytes into unactivated (M0) macrophages, phorbol 12‐myristate 13‐acetate (PMA) was applied at 320 nM for 16 h. For M1 polarization, 100 ng/mL of lipopolysaccharide (LPS) and 100 ng/mL of recombinant human interferon‐γ (IFN‐γ) were applied for 48 h. For M2 polarization, 40 ng/mL of recombinant human interleukin‐4 (IL‐4) and 20 ng/mL of recombinant human IL‐13 were applied per our well‐established protocols [38]. After confirmation of differentiation of THP‐1‐derived macrophages, we printed macrophages and synovial mesenchymal stem/progenitor cells (syMSCs), layer‐by‐layer, via Gelatin Methacryloyl (GelMA) bioink (Figure 2B). The bioprinted synovial membrane layered with M0 and syMSCs maintained the multi‐layered structure while macrophages underwent polarization (Figure 2C,D). When the M0/syMSC membrane was stimulated by M1 or M2 stimulants for 48 h, M1/syMSC and M2/syMSC secreted corresponding pro‐ (TNF‐α, IL‐1β) and anti‐inflammatory cytokines (IL‐10), respectively, at a similar level to M1‐ or M2‐printed in gel without syMSCs (Figure 2D). The cells in the bioprinted synovium showed no sign of apoptosis for up to 2 weeks (>99% viable) (Figure S3).

### 2.3 Engineering Fat

Fat was engineered by embedding 2M/mL human adipose tissue‐derived mesenchymal stem/progenitor cells (ADSCs) (ATCC, Manassas, VA) in a 5 × 3 cm^2^ disk‐shaped collagen gel (5 mg/mL) reinforced with a 3D‐printed polycaprolactone (PCL) scaffold, followed by culturing in adipogenic differentiation media as per our previous work [39]. After up to 8 weeks of culture, mature adipose tissue was engineered (Figure 2F,G). Bulk RNA‐seq was performed to validate the expression of joint fat‐enriched genes in the engineered adipose tissue. Genes highly expressed in human infrapatellar fat pad (n = 3; >50 yrs‐old male & female; no history of joint injury or OA) were highly expressed in the engineered fat tissue for 8 weeks (Figure 2H).

### 2.4 Meniscus Injury and Healing Under Inflammation and Multi‐Tissue Interactions

**FIGURE 3:**
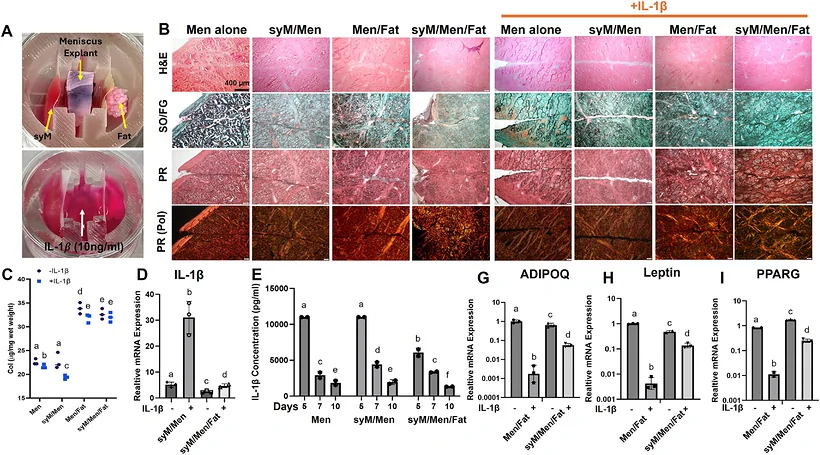
Effect of multi‐tissue interactions on meniscus healing and its response to inflammation. MoC with meniscus explant healing model, syM, and engineered fat tissue underwent bioactive glue‐mediated healing under IL‐1β stimulation (A). By 4 weeks, the meniscus healed with integration of the tissue gap regardless of co‐culturing with other joint tissues (B). However, under IL‐1β stimulation, the meniscus showed poor healing with and without syM. Interestingly, addition of fat tissue significantly mitigated the harmful effect of IL‐1β stimulation, resulting in integrated meniscus healing (B). Quantitative collagen contents at healing zone show significant increase in the groups containing fat tissue, with less reduction by IL‐1β (C). IL‐1β mRNA expression (D) and concentration (E) were significantly reduced in the groups containing fat tissue. ADIPOQ, leptin, and PPARG expressions were significantly reduced by IL‐1β in fat tissues co‐cultured with meniscus, but certain levels of these expressions were maintained even under IL‐1β when syM was included in the MoC (G‐I) (n = 3 – 5 per group; *p*<0.001; different letters indicate significant differences).

Following our established procedure [11, 12], longitudinal tears were created in the inner avascular zone of wedge‐shaped human meniscus explants (n = 3, healthy, 56–64 years old; 2 males and 1 female; no OA). Then we applied bioactive glue, FibGen + 100 ng/mL CTGF and poly(lactic‐co‐glycolic acids) (PLGA) microspheres (µS)‐encapsulating TGFβ3 (10 mg µS/mL; final concentration) [10, 11, 12]. The meniscus explants were placed on top of monolayer‐cultured human syMSCs (2 M/mL), allowing cell migration [40]. After 1 week of culture, meniscus tissues were transferred to the MoC model and secured into the PDMS mold, where syM and the engineered fat tissue were placed in the respective compartments (Figure 3A). The MoC was then cultured for 4 weeks in media with fibrochondrogenic supplements per our prior methods [10, 40], with and without 10 ng/mL of IL‐1β. As comparison groups, meniscus alone (Men), syM with meniscus (syM/Men), meniscus with fat (Men/Fat), and all three tissues (syM/Men/Fat) were included.

By 4 weeks, meniscus injuries treated with bioactive glue with syMSCs were integrated and healed regardless of co‐culture with syM and fat tissues (Figure 3B). In contrast, IL‐1β treatment disrupted meniscus healing in the meniscus alone and meniscus with syM, exhibiting remaining tissue gaps (Figure 3B). Interestingly, the meniscus co‐cultured with fat tissue healed with closed tissue gaps even under IL‐1β stimulation (Figure 3B). Similarly, all three tissue groups showed integrative meniscus healing under IL‐1β stimulation (Figure 3B). Consistently, collagen content at the healing zone was significantly lower with IL‐1β stimulation in the meniscus alone and meniscus with syM (Figure 3C). Meniscus with fat and all three tissues resulted in significantly higher collagen content at the healing zone in comparison with meniscus alone and meniscus with syM, which was significantly less dependent on IL‐1β stimulation compared to the groups without fat tissue (Figure 3C). The mRNA expression of IL‐1β in syM was significantly higher with 24 h of IL‐1β stimulation, which was significantly attenuated when fat tissue was included in the MoC (Figure 3D). Similarly, IL‐1β concentrations measured in the media after 5 days of IL‐1β stimulation were lower when fat tissue was included in the MoC compared to the groups without fat (Figure 3E). These findings suggest the anti‐inflammatory function of the engineered fat tissue. Then, the effects of IL‐1β stimulation and multi‐tissue co‐cultures were examined by the expression levels of adipokines and adipogenic markers. IL‐1β stimulation significantly reduced the expression of adiponectin (ADIPOQ), leptin, and Peroxisome Proliferator‐Activated Receptor Gamma (PPARG) in fat tissues co‐cultured with meniscus (Figure 3G–I). Interestingly, addition of syM in the MoC significantly attenuated the IL‐1β‐reduced adipokines and adipose markers (Figure 3G–I), suggesting the three‐way interactions between syM, meniscus, and fat in response to the inflammatory cytokine, IL‐1β.

### 2.5 Combinational Effect of IL‐1β and Mechanical Loading on Meniscus Healing and Joint Degeneration

**FIGURE 4:**
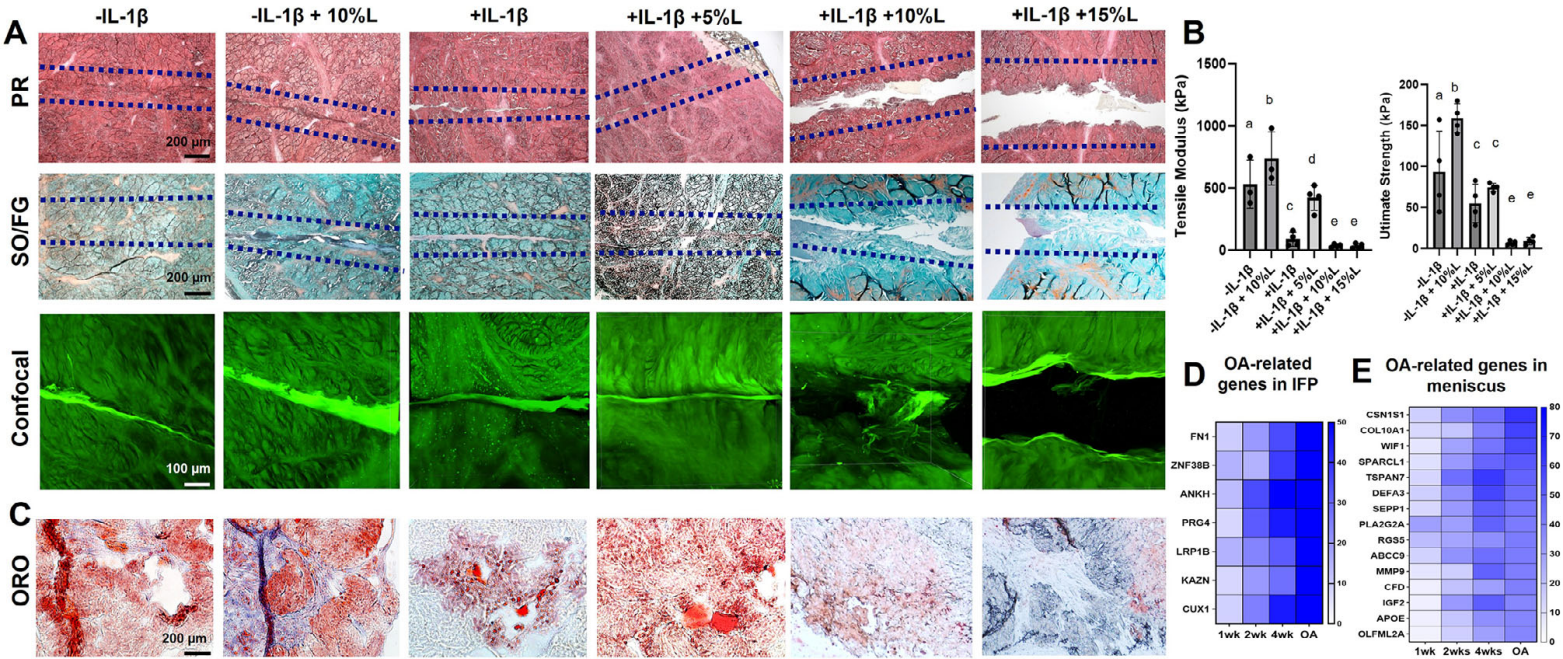
Effects of mechanical loading and IL‐1β stimulation in meniscus healing and joint degeneration. Histology with picrosirius red (PR), safranin O/fast green (SO/FG), and confocal images of optically cleared meniscus labeled with COL‐F demonstrate that mechanical loading improves meniscus healing without IL‐1β, but it can be detrimental with IL‐1β (A). Only 5% loading enhanced integrated meniscus healing under IL‐1β stimulation (A), consistent with the quantitative mechanical properties (B) (n = 3 – 4 per group; p<0.001; different letters indicate significant differences). ORO staining shows fibrosis of the engineered fat tissue under IL‐1β stimulation and 10% & 15% mechanical loading (C). RNA‐seq analysis shows OA‐related genes in human patient‐derived IPFP and meniscus are elevated in the meniscus and the engineered fat over time under IL‐1β stimulation and 10% mechanical loading (D).

We applied physiological loading to meniscus explants as a 10% dynamic femoral compression (1Hz, 30 mins/day, and 3 days/week; starting at 2 weeks after repair) using our meniscus‐specific bioreactor, per our prior work [36]. The femoral compression was applied in the perpendicular direction to the articular surface of the meniscus, which distributes into compression, tension, shear, and hoop strain, reminiscent of physiological loading applied to the knee meniscus, as described in our recent publication [36]. After 4 weeks of culture (2 weeks of loading), meniscus harvested from MoC showed integrated healing of tears without IL‐1β (Figure 4A). Application of 10% femoral compression appears to promote meniscus healing, featured by a dense collagenous matrix formed at the healing zone per confocal image of tissue‐cleared meniscus with COL‐F labeling per our prior methods [36] (Figure 4A). Interestingly, application of 10% and 15% loading in combination with IL‐1β led to deterioration of the meniscus, with enlarged tissue gap and loss of matrix (Figure 4A). In contrast, 5% loading promoted meniscus healing with enhanced tissue integration compared to 10% and 15% loading and IL‐1β without loading (Figure 4A). Quantitatively, tensile modulus and ultimate strength were significantly increased by 10% loading without IL‐1β, and only 5% loading, not 10% and 15% loading, increased the tensile properties with IL‐1β stimulation (Figure 4B). This finding may indicate that beneficial effects of mechanical loading in meniscus healing depend on the magnitude and inflammatory environment. The induced inflammation in combination with mechanical loading not only resulted in meniscus deterioration but also fibrosis of adipose tissue (Figure 4C). IL‐1β stimulation for 4 weeks led to loss of lipid droplets in the engineered fat tissue, replaced by fibrous tissue, which was more severe with 10% and 15% mechanical loading (Figure 4C). As IPFP fibrosis is one of the hallmarks of OA, these data suggest that our MoC model can replicate OA initiation from meniscus injury. Moreover, RNA‐seq analysis confirmed the increases in the OA‐associated genes in the engineered fat and meniscus over time under IL‐1β stimulation and 10% mechanical loading, comparable to the gene expression profiles from human patients with OA (Figure 4D).

### 2.6 Single‐Cell RNA‐Sequencing and CellChat Analysis Elucidating Cell‐Cell Communications in Meniscus Healing Under Inflammation and Mechanical Loading

For a comprehensive analysis of cell‐cell communication, we performed single‐cell RNA sequencing (scRNA‐seq) with Next‐Generation Sequencing (NGS), followed by CellChat analysis [41, 42, 43]. CellChat is a recently developed tool that quantitatively infers and analyzes intercellular communication networks from scRNA‐seq data. CellChat predicts primary signaling inputs and outputs for cells and how those cells and signals coordinate for functions using network analysis and a machine learning (ML)‐based pattern recognition algorithm [41, 42, 43]. All cells were isolated from the three tissues co‐cultured in the MoC model after 2 weeks, while the meniscus underwent healing induced by bioactive glue. UMAPs visualized multiple types of cells, including fibrochondrocytes, adipocytes, macrophages, and syMSCs (Figure S4A). A circle plot from CellChat analysis showed intense cell‐cell interactions between adipocytes, macrophages, and syMSCs (Figure S4B). Chord plots displayed signaling pathways involved in the cell‐cell communication between different cell types (Figure S4C).

**FIGURE 5:**
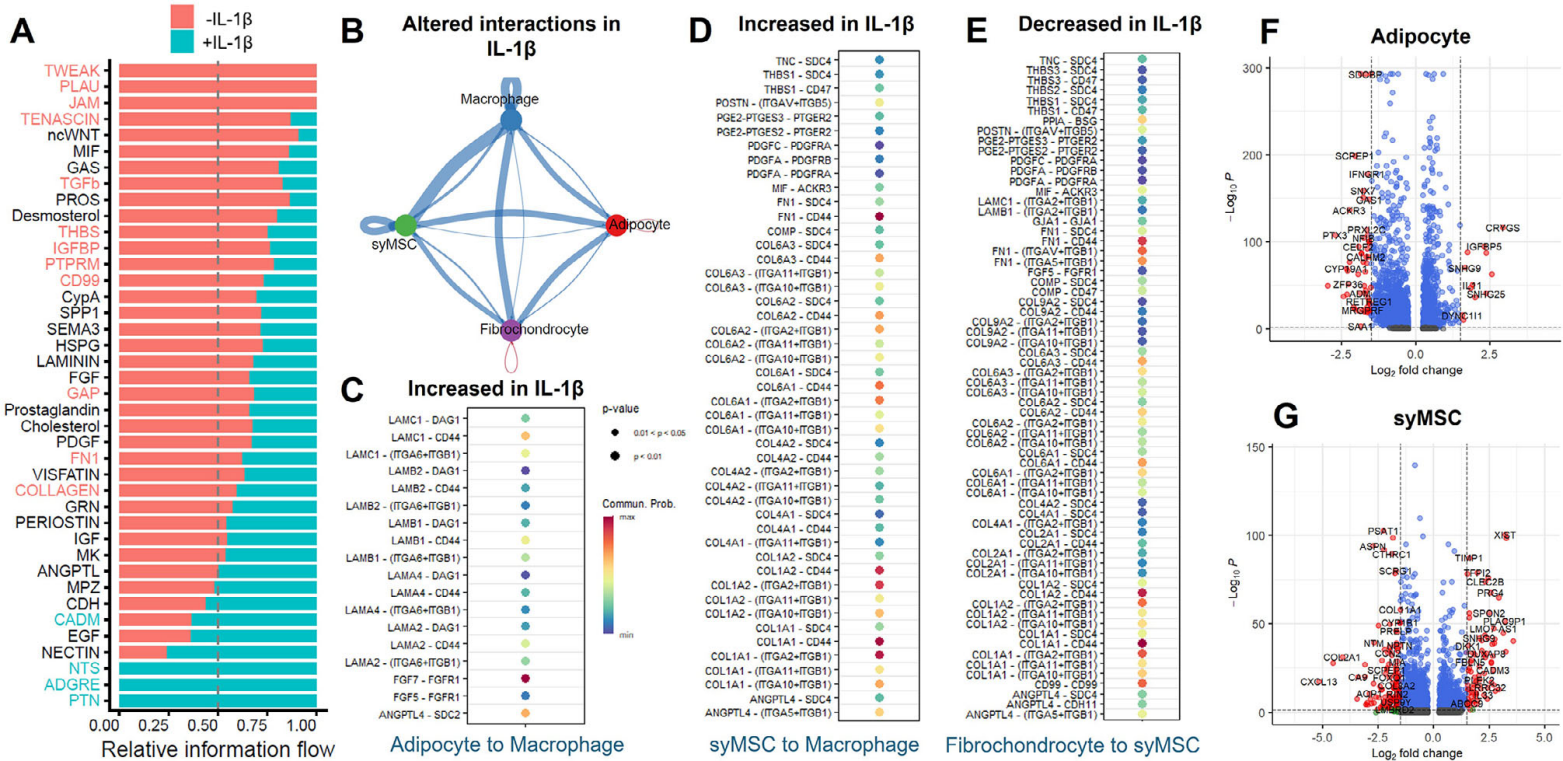
Comparative CellChat analysis for MoC without and with IL‐1β treatment. Relative information flow shows signaling increased and decreased in the IL‐1β treatment (A). The circle plot shows the altered intensities of cell‐cell interactions (B). Bubble plots show significantly increased or decreased ligand‐receptor pairs in adipocyte‐to‐macrophage (C), syMSC‐to‐macrophage (D), and fibrochondrocyte‐to‐syMSC (E). Volcano plots show DEGs in adipocytes (F) and syMSCs (G) between the IL‐1β treatment and control without IL‐1β. Threshold lines in volcano plots: |log_2_ fold change| > 1.5 and adjusted p < 0.05.

For quantitative comparisons in the cell‐cell communications with and without IL‐1β, we performed a comparative CellChat analysis. The quantitative comparison of the signal flows showed decreases in matrix metabolism‐associated genes (e.g., TENASCIN, TGFβ, FGF, FN1, Collagen, and PDGF) with IL‐1β stimulation (Figure 5A). A circle plot was created to show altered cell‐cell communication intensities in the IL‐1β‐treated group as compared to the control without IL‐1β (Figure 5B). Notable changes in cell‐cell interactions were identified between macrophage, syMSC, adipocyte, and fibrochondrocyte (Figure 5B). To describe the statistically significant changes in specific ligand‐receptor pairs, bubble plots were created (Figure 5C–F). In adipocyte‐to‐macrophage, *ANGPTL4‐SDC2*, and *LAMC‐CD44* axes showed the highest increases with IL‐1β treatment (Figure 5C). As these signaling pathways are closely related to M2 polarization of macrophages, this finding may suggest that adipocytes perform anti‐inflammatory functions by regulating macrophage polarization. In addition, differentially expressed genes (DEG) analysis in macrophages showed downregulation of early inflammatory pathways (IL6, CXCL8, and SOCS3) (Figure S5A), and GO enrichment analysis in macrophages showed upregulation of ribonucleotide biosynthesis process and oxidative phosphorylation, closely associated with M2 polarization [44, 45] (Figure S5B). *FGF7‐FGFR1* axis was also identified as a significantly increased ligand‐receptor pair, but it is likely a false positive, as FGF7 specifically binds FGFR2‐IIIb in epithelial cells [46, 47, 48], while macrophages express FGFR1‐IIIc [47]. This is likely due to limited specificity of the current CellChat ligand‐receptor database for FGFR isoforms. Moreover, FGF7 is not a secreted protein from adipocytes although its gene is expressed in adipocytes [49]. Thus, we omitted the FGF7‐FGFR1 for further analysis of adipocyte‐to‐macrophage interactions.

### 2.7 Comparative CellChat Analysis Between With and Without Mechanical Loading

A comparative CellChat analysis was performed to delineate the roles of cell‐cell communication involved in the enhanced meniscus healing by 5% loading under IL‐1β treatment. Similarly with the other groups, MoC with 5% loading and IL‐1β showed adipocytes, fibrochondrocytes, syMSC, and macrophages, with active interactions between the cell types (Figure S6).

**FIGURE 6:**
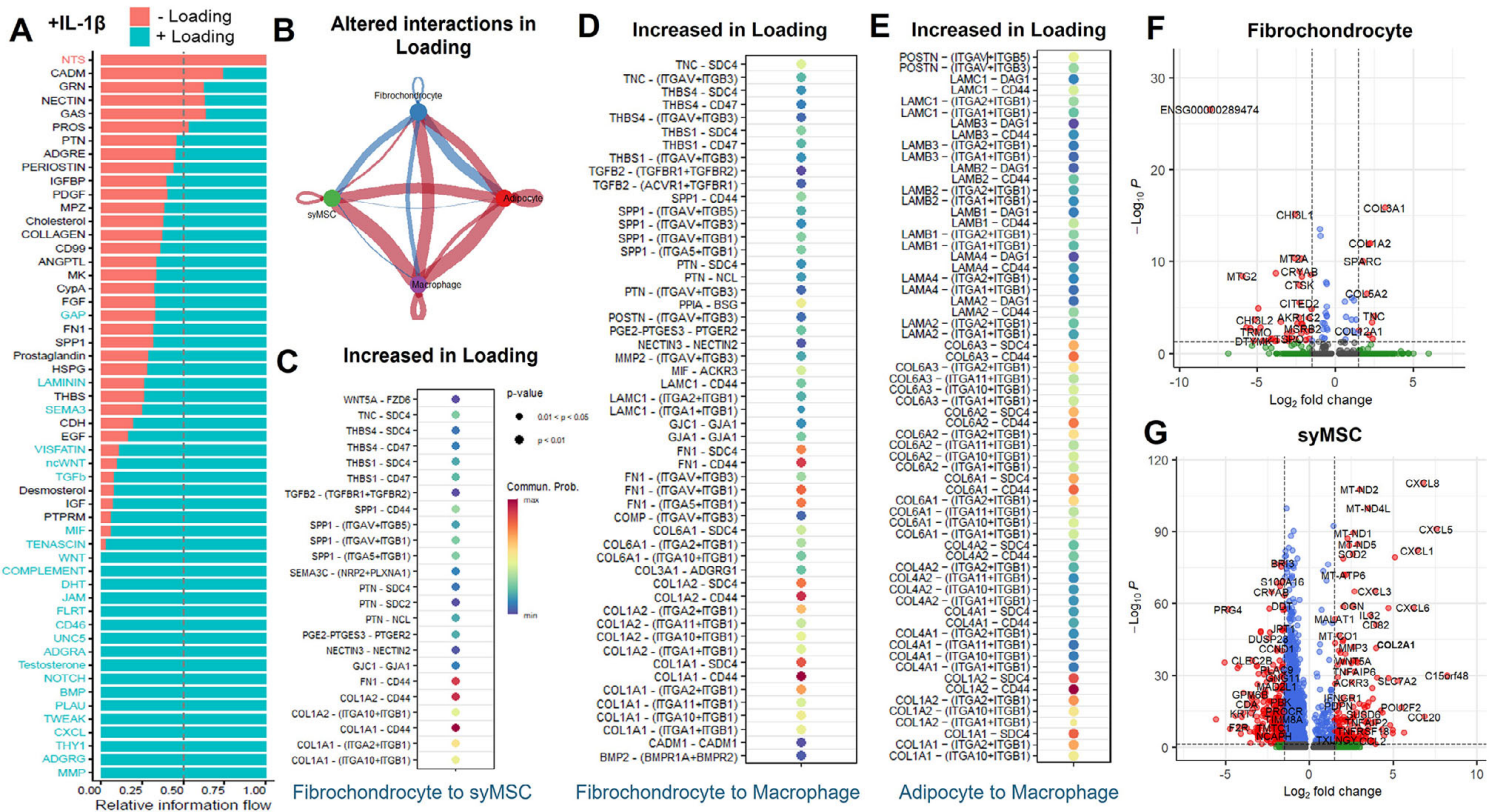
Comparative CellChat analysis for MoC under L‐1β treatment with and without mechanical loading. Relative information flow shows signaling increased and decreased by mechanical loading (A). The circle plot shows the altered intensities of cell‐cell interactions by mechanical loading (B). Bubble plots show significantly increased ligand‐receptor pairs in fibrochondrocyte‐to‐syMSC (C), fibrochondrocyte‐to‐macrophage (D), and adipocyte‐to‐macrophage (E). Volcano plots show DEGs in fibrochondrocyte (F) and syMSC (G) between the MoC without and with mechanical loading. Threshold lines in volcano plots: |log_2_ fold change| > 1.5 and adjusted p < 0.05.

Application of 5% dynamic physiological loading increased signals associated with matrix synthesis and differentiation, such as HSPG, TGFβ, TENASCIN, BMP, and FGF (Figure 6A). The circle plot showed notably altered cell‐cell interactions by mechanical loading compared to MoC without loading (Figure 6B). Specifically, signals related to fibrochondrogenic differentiation (e.g., fibronectins and collagens) were significantly elevated by mechanical loading (Figure 6C). Collagen and fibronectin signals were also significantly elevated in fibrochondrocyte/adipocyte‐to‐macrophage (Figure 6D,E). Consistently, volcano plots showed significant increases in fibrochondrogenic matrix synthesis‐related genes (e.g., *COL1A1, COL1A2, TNC*, *COL2A1*, and *SPARC*) (Figure 6F). In syMSC, both chemokine genes (e.g., *CXCL1, CXCL5, CXCL6, CXCL8*, and *CCL20*) and differentiation/regeneration‐associated genes (e.g., *COL2A1, COMP*, and *IL32*) were significantly increased by mechanical loading (Figure 6G).

In contrast to +IL‐1β vs. ‐IL‐1β, mechanical loading reduced the ANGPL4‐SDC2 axis (Figure S7A), suggesting the loading‐enhanced healing may not be directly associated with anti‐inflammatory signals from adipocytes toward macrophages. Regardless, mechanical loading significantly reduced pro‐inflammatory genes (e.g., *CXCL1, CXCL5, CXCL6, CXCL12, MMP13, MMP8, HSD11B1, and IDO1*) in adipocytes and elevated regeneration/protection‐related genes (e.g., *FGF18, PRELP*, and *MGP*) (Figure S7B). Consistently, pro‐inflammatory genes in macrophages were significantly reduced by mechanical loading (Figure S7C). Given the decreased ANGPL4‐SDC2 signal from adipocyte to macrophage, it is suggested that different cell‐cell communication signals may be involved in anti‐inflammatory traits in macrophages with mechanical loading. One candidate is the mechanical loading‐increased collagen and fibronectin signals from fibrochondrocytes to macrophages, as *COL1A1, COL1A2*, and *FN1* genes are positively correlated with M2 polarization [50, 51, 52].

To sum, our comparative CellChat analysis suggested that mechanical loading of the meniscus stimulates fibrochondrogenic differentiation of fibrochondrocytes and syMSCs, leading to enhanced meniscus healing. In addition, collagen and fibronectin signals from fibrochondrocytes and adipocytes to macrophages may be linked with anti‐inflammatory shifts in macrophages by mechanical loading.

### 2.8 Comparative Analysis Between Intact and Defect Only

As our meniscus explants were prepared by cutting the whole meniscus into wedge‐shaped tissue pieces, we performed a comparative scRNA‐seq analysis between MoC with intact meniscus and defect alone (longitudinal tears created in the prepared explant with no treatment) to confirm the biological activities caused by defects in meniscus explants. After 2 weeks of culture with untreated defects in the meniscus explants, the macrophage cluster disappeared, and a synovial fibroblast cluster was identified instead (Figure S8A). However, the intact group exhibited fibrochondrocyte, syMSC, adipocyte, and macrophage clusters, the same as the other treatment groups (Figure S8D). The defect‐only samples showed relatively low intensities of cell‐cell interactions compared to the other treatment groups (Figures S4B,E, and S6B), except for the interactions between syMSC and synovial fibroblasts (Figure S8B,C). The intact group showed active interactions between adipocytes, macrophages, and fibrochondrocytes (Figure S8E,F).

DEG analysis revealed large numbers of genes significantly increased and decreased in syMSCs, adipocytes, and fibrochondrocytes in the defect only, as referenced to the intact (Figure S8A). To better describe the biological activities based on these altered gene expressions, we performed Pathway Responsive GENes for activity inference (PROGENy) analysis [53] between the defect alone and intact, weighting the top 500 downstream target genes for 14 core signaling pathways. As a result, the defect alone showed high activity scores in EGFR, MAPK, NF‐κB, TNF‐α, p53, and Trail pathways (Figure S9B). Given the roles of these pathways in tissue repair signals (EGFR) [54], pro‐inflammation (MAPK, NF‐κB, and TNF‐α) [55, 56], and cell death (p53 and Trail) [57, 58, 59], these data demonstrated the clear biological distinction created by a full‐thickness longitudinal tear in the wedge‐shaped meniscus explant in the MoC model.

### 2.9 Comparative Analysis Between the Defect Alone and Bioactive Glue Treatment

We performed a comparative analysis between the defect and bioactive glue treatment to determine the effects of bioactive glue treatment on the detrimental signaling pathways observed in the defect alone in MoC. The overall cell‐cell communication signal flow showed increases in matrix metabolism/cartilage protection‐associated pathways in bioactive glue‐treated MoC, including HSPG, PDGF, TGFβ, EGF, CADM, FGF, and IGF (Figure S10A). In fibrochondrocytes and syMSCs, genes related to fibrochondrogenic differentiation and matrix formation (e.g., *COL2A1, SOX9, COL1A2*, and *CHAC1*) were significantly elevated by bioactive glue treatment, in comparison with the defect alone (Figure S10B). These findings support the expected functions of bioactive glue promoting meniscus healing by activating fibrochondrocytes and syMSCs.

To evaluate whether adipocyte‐derived anti‐inflammatory signals were from systemic cell‐cell interactions or a direct effect of TGFβ3 released from bioactive glue, we performed PROGENy analysis for adipocytes alone between the bioactive glue and defect groups (Figure S10B). As expected from the other dataset, the activity scores for inflammatory pathways (e.g., NF‐κB and TNF‐α were notably decreased with bioactive glue treatment (Figure S10B). However, MAPK pathways showed a very low activity score. As MAPK is a direct downstream of the TGFβ3 pathway [40, 60], this observation suggests that adipocyte‐derived anti‐inflammatory functions are likely indirect and systemic, rather than a direct effect of TGFβ on adipocytes.

### 2.10 *V*alidation of Anti‐Inflammatory Role of ANGPTL‐4 From Adipocytes

We conducted experiments to confirm the CellChat‐identified anti‐inflammatory function of ANGPTL4 toward macrophages. First, the concentration of ANGPTL‐4 in the tissue culture chamber was significantly elevated when three tissues were co‐cultured under IL‐1β, consistent with its increased signal under IL‐1β (Figure S11A). When applied to macrophages, ANGPTL‐4 (200 ng/mL) significantly reduced M1 polarization markers and increased M2 markers by 24 h of treatment with respective polarization stimulations (Figure S11B–E).

### 2.11 Comparison With Human Data

To assess the clinical relevance of the identified cell‐cell interactions, we compared our MoC CellChat data with human data. Integrated scRNA‐seq data were created from 7 healthy human menisci (GSE220243) and 3 sets of synovium and fat pad (GSE226651) per a previously described *HarmonyIntegration* method [61]. Similarly, human OA data were created from 7 OA menisci (GSE220243) and 3 OA synovium & fat pads (GSE226651) [61]. Integrated scRNA‐seq data were used to perform CellChat analysis, followed by comparison with CellChat objects from MoC. As a result, the MoC with intact meniscus showed 1,032 matched interactions (p<0.000001) with healthy human data, showing various interaction intensities (Figure S12A). MoC with meniscus injury and IL‐1β stimulation showed 470 matched interactions (p<0.000001) with human OA data, showing a range of interaction intensities (Figure S12B).

## 3 Methods

### 3.1 Design and Construction of the MoC

#### 3.1.1 Main Chip

MoC was constructed as a polydimethylsiloxane (PDMS) chamber with three compartments separated by a semi‐permeable nylon membrane with 20 µm pores. The PDMS structure was fabricated using a 3D‐printed PLA negative mold designed with a commercialized computer‐aided design (CAD) program (CATIA R19, Dassault Systèmes). Silicone elastomer and curing agent were mixed at a 10:1 weight ratio, then degassed using a vacuum chamber for >15 mins to remove trapped air bubbles. Then, the degassed silicone was poured slowly into the prepared molds and placed in a 60°C oven for 6 h to promote uniform cross‐linking and optimize the mechanical properties of the silicone. After curing, the PLA negative mold and cured PDMS body were carefully extracted to avoid structural damage. The treated surfaces were aligned and pressed together immediately post‐treatment.

#### 3.1.2 Synovium Membrane (syM)

Using a custom‐built, multi‐channel bioprinter, human THP‐1 cell‐derived macrophages were printed via our gelatin‐based bioink [37], then P2 – 3 human synovial MSCs were printed on top of the macrophage lining. THP‐1 human monocytes (ATCC, Manassas, VA) were cultured in complete RPMI media (ThermoFisher Scientific, Waltham, MA), supplemented with 10% heat‐inactivated FBS. For differentiation of THP‐1 monocytes to unactivated (M0) macrophages, phorbol 12‐myristate 13‐acetate (PMA) was applied at 320 nM for 16 h. For M1 polarization, 100 ng/mL of lipopolysaccharide (LPS) and 100 ng/mL of recombinant human interferon‐γ (IFN‐γ) were applied for 48 h. For M2 polarization, 40 ng/mL of recombinant human interleukin‐4 (IL‐4) and 20 ng/mL of recombinant human IL‐13 were applied as per our well‐established protocols [38]. To confirm M1 and M2 polarization in syM, ELISA was conducted for TNF‐α, IL‐1β, and IL‐10. Live/Dead assay (Thermo Fisher Scientific, Waltham, MA, USA) was conducted for syM after 2 weeks in vitro. Briefly, bioprinted syM was washed with phosphate‐buffered saline (PBS) and incubated with a staining solution containing calcein‐AM (5 µl) and ethidium homodimer‐1 (20 µl) in 10 mL PBS for 30 min at 37°C in an incubator. After staining, syM was gently washed with PNS and imaged using a fluorescence microscope (excitation/emission: 488/515 nm for live cells and 528/617 nm for dead cells).

#### 3.1.3 Engineering Fat Tissue

Fat tissues were engineered by embedding 2M/mL of P2‐3 human adipose tissue‐derived mesenchymal stem/progenitor cells (ADSCs) (ATCC, Manassas, VA) in a 5 × 3 cm^2^ disk‐shaped collagen gel (5 mg/mL) reinforced with a 3D‐printed PCL scaffold, followed by culturing in adipogenic differentiation media as per our previous work [39]. By 8 weeks of culture, the maturity of engineered fat tissues was evaluated using Oil Red O staining of frozen tissue sections.

### 3.2 RNA‐Seq

RNA‐seq was conducted to validate the engineered fat tissue for the expression of joint fat‐enriched genes in the engineered fat. Briefly, human infrapatellar fat pad (IPFP) tissues were obtained from donors with no history of joint injury or osteoarthritis (OA) through the National Disease Research Interchange (NDRI) (IRBexempt under 45 CFR 46). This is included in the methods section. Tissues were gently washed with PBS and then finely minced and enzymatically digested in Dulbecco's modified Eagle's medium (DMEM) containing collagenase type I (5 mg/mL), collagenase IV (1 mg/mL) at 37°C for 30 min with gentle agitation. The digested tissue was filtered through a 70 µm cell strainer to remove undigested debris. Cells were washed with PBS and immediately processed for RNA extraction. Total RNA was isolated using a commercial RNA extraction kit according to the manufacturer's instructions (QIAGEN, RNeasy Mini Kit, Hilden, Germany). RNA concentration and purity were assessed using a spectrophotometer (NanoDrop, Thermo Fisher Scientific, Waltham, MA, USA), and RNA integrity was evaluated an Agilent Bioanalyzer (Agilent Technologies, Santa Clara, CA, USA). Samples with an RNA Integrity Number (RIN) greater than 7.0 were used for downstream analysis. RNA‐seq data from the engineered fat was compared with human adipose tissues (NDRI; n = 3; >50 yrs‐old male & female; no history of joint injury or OA).

RNA‐seq was also performed to determine OA‐related gene expression profiles in the engineered fat and meniscus explant under IL‐1β and mechanical loading. Human RNA‐seq data from OA patients were used for the comparisons (GSE45233 from 4 adult healthy menisci; <40 years old; 3 males and 1 female). OA patients' derived IPFP was analyzed using RNA‐seq (NDRI, n = 2; 53–65 years old; 1 male and 1 female).

### 3.3 Meniscus Bioreactor and Loading Application

To apply physiological loadings to the meniscus, we developed a meniscus‐specific bioreactor (430 × 300 × 500 mm^3^) fitting in a CO_2_ incubator. The bioreactor system is equipped with 3‐axis motion control units, and the range of the stroke is 150 mm, 150 mm, and 50 mm in x‐, y‐, and z‐axis, respectively, with a 100 µm resolution in the location control. The entire loading unit is built‐in with a chamber enabling humidity and CO_2_ level control. All the parts, including the stimulation part holder, stimulation part, 6‐well plate holder, meniscus explant holder, and stimulation part, were fabricated with polylactic acid (PLA) by conventional 3D‐printing. The movements in 3‐axis are controlled by a custom‐programmable G‐code that manages x‐, y‐, and z‐axis actions, consequently leading to the application of various motions, including compression, tension, and shear on the meniscus. Details on the meniscus bioreactor is described in our recent publication [36]. Femoral compression loading (5% – 15%) was applied through a femoral condyle‐shaped loading unit in the perpendicular direction of articular surface of meniscus explants. The strain percentage was calculated as compressive displacement per radial thickness of the meniscus explant from the surface to the bottom corner, as described in our recent publication [36]. A pre‐determined loading frequency and duration were applied (1Hz for 30 mins/day, 3 days/week). The loading was applied starting at 2 weeks after meniscus repair, as a predetermined loading regime to promote meniscus healing.

### 3.4 Cytokine Assays

A multiplex immunoassay kit (ProcartaPlex, ThermoFischer) was used to measure cytokines and growth factors involved in inflammation and immune responses, including but not limited to IL‐1β, IL‐10, IL‐10, and TNF‐α. The assay was performed according to the manufacturer's instructions. Briefly, collected culture media from the tissue compartment were centrifuged to remove debris, and 25 µL of the supernatants were used for the assay. Samples were incubated with magnetic beads conjugated with capture antibodies in a 96‐well plate, followed by sequential incubation with detection antibodies and streptavidin‐PE. The beads were analyzed using Luminex, and cytokine concentrations were calculated from standard curves generated with known concentrations.

### 3.5 Meniscus Injury Healing Explant Model

We prepared wedge‐shaped meniscus explants as described in our prior works [12, 40, 62, 63, 64]. Briefly, menisci were isolated from human knee joints (NDRI; n = 3, healthy, 56–64 years old; 2 males and 1 female; no OA). The inner third zone of the menisci was cut from the central region, avoiding the horn area, and prepared as wedge‐shaped tissue explants in a thickness of 5 mm, followed by creating full‐thickness, longitudinal incisions in the middle of the inner third zone. Then the defect was applied with bioactive glue, fibrin gel crosslinked with genipin (FibGen) (100 mg/mL fibrinogen, 100 U/mL thrombin, and 2.5 mg/mL genipin) loaded with connective tissue growth factor (CTGF) (BioVendor, Asheville, NC) (100 ng/mL; final concentration) and poly (lactic‐co‐glycolic acids) (PLGA) microspheres (µS)‐encapsulating TGFβ3 (10 mg µS/mL; final concentration). The repaired meniscal explants were cultured in 6‐well tissue culture plates, suspended with human syMSCs (1 × 10^6^ cells/mL) for a week. Then, the meniscus explants were secured in the MoC. After harvesting the tissues, quantitative and qualitative analyses were performed, including qRT‐PCR, collagen assay at the healing zone, histology, mechanical testing, and scRNA analysis as described below and in our prior works [10, 40, 64].

### 3.6 qRT‐PCR

Total RNA was extracted using the Qiagen RNAeasy mini kit (#74104, Qiagen, Germany) according to the manufacturer's instructions. RNA concentration and purity were determined using a NanoDrop spectrophotometer. A 500 ng RNA was used to synthesize complementary DNA using a reverse transcription reagents kit (#433182, Thermo Fisher, Waltham, MA, USA). The real‐time quantitative PCR was conducted with a ViiA7 real‐time system (Thermo Fisher, Waltham, MA, USA).

### 3.7 Histology

The collected tissues were fixed in 4% paraformaldehyde, embedded in paraffin, and sectioned at 5‐µm thickness. The tissue sections were then subjected to xylene treatment to eliminate the paraffin, followed by a stepwise rehydration process before conducting specific staining, including H&E, Safranin‐O (Saf‐O), and Picrosirius Red (PR) according to our established protocols [12, 40, 63]. All histology images are representative samples of >10 randomly selected tissue sections per group.

### 3.8 Tissue Clearing of Meniscus Explant for 3D Volumetric Analysis of Collagen Orientation

To evaluate collagen fiber organization throughout the tissue depth, we performed optical clearing of meniscus tissues, aimed at reducing light scattering and absorption, thus allowing deeper light penetration. As described in our recent publication [36], we implemented a modified THF (Tetrahydrofuran)‐DBE (Dibenzyl ether) method [65] that we optimized for meniscus tissues. To label collagen fibers, Col‐F, a collagen‐binding green fluorescence probe (ImmunoChemistry Technologies, LLC, Bloomington, MN) was prepared by dissolving it in DMSO to a stock concentration of 20 mm, followed by a working concentration of 15 µm in PBS. Meniscus explants were prepared as 1 mm‐thick sections and stained by submerging in 300 µL of a 15 µM Col‐F working solution for 1 h at 37°C. Following the staining procedure, the meniscus tissues were dehydrated in 50% THF solvent overnight, then subjected to progressively increased concentrations of THF (10% increments, up to 90%) for 1 h at each concentration to prevent tissue contraction. After dehydration in 90% THF, the tissue was fully dehydrated in 100% THF solvent overnight. Finally, the dehydrated meniscus explants were submerged in DBE solution overnight for optical clearing. Optically cleared meniscus samples with Col‐F labeling were examined by a confocal microscope (Red AXR/Ti2, Nikon Americas Inc., Melville, NY) for 3D volumetric collagen fiber orientations.

### 3.9 Mechanical Test

Tensile tests were performed for the harvested meniscus explant samples as per our prior work (12). The samples for the tensile tests were prepared as 8 mm in length, 2 mm in width, and 1 mm in thickness. Upon mounting with tensile jigs in an isotonic saline bath at RT, a 0.02‐N tare load was applied to the samples, and then the samples were elongated at 10%/min until failure using uniaxial mechanical testing (CellScale, Waterloo, ON, Canada). From the stress–strain curve, tensile modulus and ultimate strength were calculated.

### 3.10 scRNA‐seq and CellChat Analysis

After 2 weeks, cells were isolated from the healing zone of the meniscus (5 mm‐thick), syM, and fat tissues. The harvested tissues were minced, followed by 5 mg/mL collagenase I in serum‐free DMEM for 2.5 h at 37°. After enzymatic digestion, an equal volume of 10% FBS in DMEM was added to neutralize the enzymes. Suspended cells were transferred to a 100 µm strainer, centrifuged at 1 600 rpm, and rinsed 3 three times using PBS with 10% FBS. Library construction and scRNA‐seq were performed on the 10XGenomics platform. The scRNA‐seq analysis was conducted in R (version 4.4.2) with a rigorously developed pipeline integrating multiple analytical tools. Raw count matrices were imported from HDF5 files, from which the Seurat objects were generated separately for each sample using Seurat (version 5.2.1). Quality control steps entailed filtering out cells with fewer than 200 or more than 6 000 detected features and with greater than 20% mitochondrial gene expression. Gene expression data were then globally normalized, and the top 2 000 variable genes were identified using a variance‐stabilizing transformation. Scaled data were subjected to principal component analysis (PCA). The number of principal components used in downstream analyses was determined via the elbow plot by identifying the inflection point at which additional principal components contributed only marginally to the variance. Doublet detection was performed using DoubletFinder (version 2.0.4) with an estimated doublet rate of approximately 7.5%, and only cells classified as singlets were retained. Following normalization and filtering, control and OP datasets were merged and batch‐corrected using Harmony (version 1.2.3) with the original sample identifier as the grouping variable. Clustering was conducted based on the optimal number of PCA components (as determined by the elbow plot) and subsequently visualized using both UMAP, produced via ggplot2 (version 3.5.1) and patchwork (version 1.3.0). Cell type annotation was initially performed via differential expression analysis using Seurat's FindAllMarkers function (with a log_2_ fold‐change cutoff of 0.25 and a minimum percentage threshold of 25% for gene expression). We used marker genes to label each cell type: fibrochondrocytes (COL1A1, COL2A1, and ACAN), macrophages (CD68), syMSC (NT5E and THY1), and adipocytes (APOE, PLPP4, and SLC40A1). These markers were distinctively expressed in the respective cell clusters in our scRNA‐seq data.

To elucidate intercellular comdmunication, a dedicated Seurat object was constructed for CellChat analysis [42] using CellChat (version 1.6.1) and associated packages (circlize version 0.4.16, grid version 4.4.2, and Matrix version 1.7.3), with a human CellChat database. Custom visualization functions were developed to generate two types of plots: a circular network plot and chord diagrams. The circular network plot displays the global cell–cell communication landscape, where vertex sizes indicate cell population sizes and edge weights reflect the strength of communication probabilities. The chord diagram is designed to illustrate specific signaling interactions, highlighting the selected signaling pathways based on interaction scores or selected pathway of interest. In these chord diagrams, chord thickness denotes the communication strength between cell types, offering insights into the key ligand–receptor mediated interactions. Quantitative comparisons of cell‐cell communication signals were performed between the control and 4‐PPBP treatment groups, using comparative CellChat analysis. Briefly, CellChat objects created from control and treatment samples were merged into a new CellChat object. The merged CellChat object was analyzed for cell‐cell communications, and significantly upregulated or downregulated cell‐cell signaling pathways were identified for each cell‐cell pair, followed by visualization with bubble plots. Differential expression analyses between the control and treatment groups within each annotated cell type were further carried out, and significant changes were visualized using volcano plots, heatmaps, and dot plots.

PROGENy analysis was performed using combined Seurat objects in each paired comparison group. Activity scores for 14 signaling pathways were calculated, and higher scores indicate higher computed pathway activation.

### 3.11 Statistical Analysis

Statistical analyses were performed using GraphPad Prism (version 10.2.3) for all quantitative data. A one‐way Analysis of Variance (ANOVA) with post‐hoc Bonferroni tests was applied with a significance level set at p < 0.05. All data are presented as means ± SD, unless stated otherwise. To ensure scientific rigor and unbiased data analysis, all qualitative and quantitative assessments were conducted in a blinded manner.

## 4 Discussion

Despite the increasing interest in multi‐tissue crosstalk involved with OA, the meniscus has been largely neglected in the equation. This study, for the first time, discovered the critical roles of three‐way crosstalk between meniscus, synovial membrane, and fat in the regulation of meniscus injury, degeneration, and healing. The simple and straightforward meniscus‐on‐a‐chip (MoC) model developed in this study successfully replicated the OA initiation and progression from meniscus injury when stimulated by inflammation and mechanical loading. Our MoC model integrated with a meniscus‐specific bioreactor enabled a comprehensive investigation of the combinatorial effects of inflammation and physiological/pathological loading on meniscus healing and degeneration. By implementing scRNA‐seq and CellChat analysis, this study discovered how communications between adipocytes, macrophages, fibrochondrocytes, and syMSCs regulate the fate of meniscus injury in response to exogenous stimulation.

Our scRNA‐seq with CellChat analysis has identified a potential cell‐cell communication signal, *ANGPTL4‐SDC2* in adipocyte‐to‐macrophage, which is robustly increased under IL‐1β stimulation when three tissues were co‐cultured. Although *FGF7* was also significantly increased in adipocytes with IL‐1β, it was excluded from our analysis of the functional cell‐cell communication signal, given that FGF7 protein is not secreted from adipocytes, in contrast to other FGF isoforms that function as secreted adipokines, such as FGF1, FGF10, and FGF21 [66, 67, 68]. Moreover, FGF7 specifically binds to FGFR‐IIIb that is exclusively expressed in epithelial cells, while macrophages express FGFR‐IIIc with negligible binding affinity for FGF7 [69]. Accordingly, ANGPTL4 is suggested as a potential cell‐cell communication signal from adipocytes to macrophages, likely reducing inflammation. Previously, the anti‐inflammatory function of ANGPTL4 in facilitating macrophage polarization was reported in cardiac repair [70], and recent spatial interactome studies in tumor niches have identified SDC2 as a high‐affinity receptor for stromal ANGPTL4, regulating immunosuppressive niches [71]. Thus, our novel discovery of the ANGPTL4‐SDC2 axis in adipocyte‐to‐macrophage may bridge these findings, suggesting its potential roles in orchestrating metabolic reprogramming and M2 polarization involved in enhanced meniscus healing under an inflammatory environment. Consistently, in vitro experiments support the anti‐inflammatory function of ANGPTL4, attenuating M1 polarization and promoting M2 polarization. Our validation experiment also confirmed that ANGPTL4 is activated when three tissues were co‐cultured under IL‐1β, likely indicating the importance of the three‐way crosstalk between meniscus, syM, and fat.

Physiologically, knee menisci are exerted by 5%–15% dynamic strain [35, 36, 72]. Thus, 10% loading has been widely adopted for research on mechano‐regulation of meniscus healing, tissue engineering, and mechanobiology [35, 36, 72]. Consistently, our study showed that 10% physiological loading of meniscus explants significantly enhanced meniscus healing, similarly with our prior work [36]. However, we observed joint tissue degeneration when 10% loading was applied to the meniscus with IL‐1β stimulation. In contrast, 5% loading enhanced meniscus healing without signs of degenerative changes, even with IL‐1β stimulation. In this context, we identified an interesting shift in the three‐way cell‐cell communication profiles regulated in meniscus healing under inflammation when 5% loading was applied. The application of 5% loading with IL‐1β reduced ANGPTL‐SDC2 in adipocyte‐to‐macrophage compared to IL‐1β without loading. Instead, signals associated with matrix synthesis were highly increased in fibrochondrocyte‐to‐syMSC communication (e.g., *COL1A1‐CD44*, *COL1A2‐CD44*, and *FN1‐CD44*), consistent with elevated gene markers for matrix synthesis and fibrochondrogenic differentiation in fibrochondrocytes and syMSCs. Accordingly, our data suggest that the loading applied to the meniscus promotes syMSC differentiation, leading to enhanced meniscus healing, consistent with the previously reported roles of mechanical stimulation in inducing MSC differentiation [73]. Interestingly, mechanical loading also resulted in the reduction of pro‐inflammatory genes in adipocytes and macrophages. Given the lack of significantly elevated anti‐inflammatory signals from fibrochondrocytes and syMSCs, as the first responders to mechanical loading, the anti‐inflammatory modulation of mechanical loading is likely an indirect effect. Although the elevated *COL1A1‐CD44* axis in fibrochondrocyte‐to‐macrophage may drive macrophages toward a pro‐remodeling phenotype [74], leading to matrix maturation and preventing excessive inflammation, we do not have experimental evidence to support the hypothesis.

Limitations of this study include the simplification applied to our joint model without cartilage, subchondral bone, and ligaments, as an incomplete joint. Our MoC is designed to investigate the effect of inflammation and mechanical loading on meniscus injury and healing, rather than focusing on OA involving cartilage degeneration. As our target is the early phase of meniscus injury and how the injury can heal or progress into degenerative changes influenced by multi‐tissue crosstalk and exogenous stimulants, we designed a simplified three‐tissue model in consideration of technical challenges to build a complex, complete Joint‐on‐a‐chip. In our knee joint, all mechanical loadings are applied to the meniscus through subchondral bone and cartilage, and factors derived from cartilage and bone may play essential roles in multi‐tissue crosstalk. Thus, the current MoC is limited in replicating a complete knee joint system. Despite the inevitable limitation, we believe that our simplified MoC, with the primary tissue components with dominant roles, may provide a reliable research platform for the scope of this study to understand three‐way crosstalk between meniscus, synovium, and fat. In addition, our MoC demonstrated the capacity to recapitulate the initiation of OA from meniscus injury without treatment, further supporting the scientific premise that synovial membrane and IPFP play central roles in post‐trauma OA [29, 30]. Our simplified three‐tissue model not only possesses a limitation but also a technical advantage, as it does not require separate microfluidic channels to provide tissue‐specific media supply. Meniscus, syM, and engineered fat maintained their respective phenotypes up to 4 weeks of co‐culture, while inducing meniscus injury healing in the simple and straightforward MoC model, although the 4‐week only captures the early phase that may not fully represent the durability of healing and prevention of fat fibrosis in the long‐term.

Another limitation of this study is the current paucity of methodology for validating the CellChat‐identified cell‐cell communication signal pathways. As previously reported [75, 76], simplified co‐culture experiments with signaling inhibitors may be explored to validate the cell‐cell communication signals. However, the interactions between different cell types are likely involved in the systemic network of multi‐tissue interactions, rather than communications between cell pairs [77, 78], resulting in technological challenges. Although our study validated the anti‐inflammatory function of ANGPTL4 in macrophage culture, it still leaves many unanswered questions on how multi‐tissue interactions guide the systemic effects. Thus, we attempted to validate the cell‐cell interactions inferred from the MoC scRNA‐seq data with the existing human scRNA‐seq data integrated from multiple human samples of meniscus, synovium, and fat with and without OA. Importantly,1 032 and 470 statistically significant interactions were matched between human data and MoC, in health joint and OA, respectively. These observations support the clinical relevance of our findings from MoC. However, several technical challenges remain. The available human scRNA‐seq data are integrated from multiple, separate datasets from different human donors, rather than from a single joint. Thus, although multiple human donor data were integrated, the inferred cell‐cell communications may contain some individual variances. Moreover, the currently available human OA data are from the late stage undergoing total knee replacements. In contrast, the induced OA in MoC may represent a relatively earlier stage initiated from meniscus injuries and inflammation. Unfortunately, there is no human data on the early stage of PTOA. Future studies can be designed to clearly describe the time sequence and cause‐and‐effect of complex communication signaling networks between synovium, meniscus, and fat, validated through human data collected at multiple time points after meniscus injury.

**FIGURE 7:**
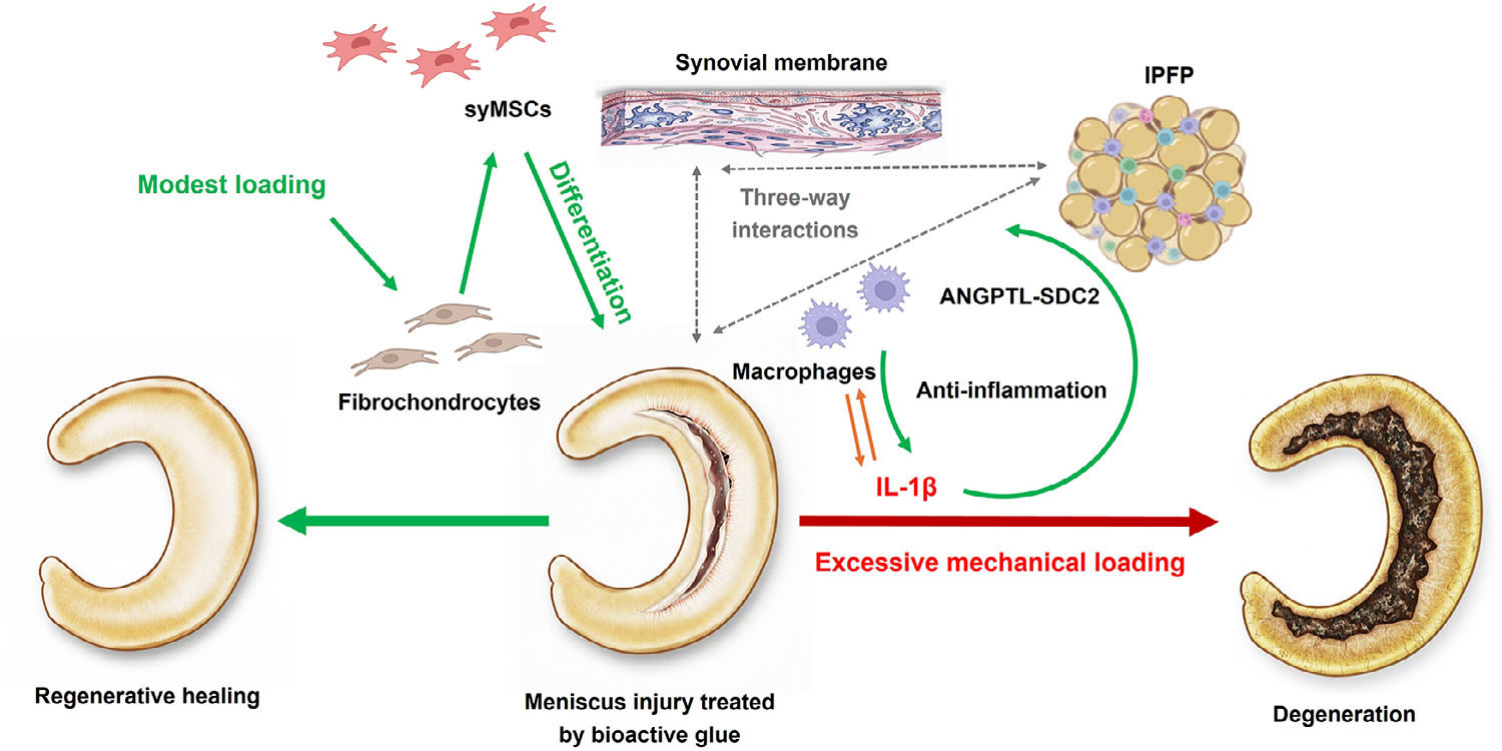
Roles of multi‐tissue crosstalk regulating meniscus healing under inflammation and mechanical loading through three‐way interactions between synovium, meniscus, and fat.

Despite those limitations, this study represents the first identification of essential cell‐cell communication signals between meniscus, synovial membrane, fat, and syMSCs, which are responsible for regulating meniscus healing under inflammation and mechanical loading (Figure 7). These novel findings may have significant implications for developing next‐generation therapeutics for meniscus injury and prevention of joint degeneration through regulating multi‐tissue crosstalk in the joint.

## Author Contributions

**HJJ**: Conceptualization, Investigation, Formal analysis, Data curation, Resources, Writing – original draft, review & editing. **EZ, MSK, MW** & **SC**: Experimental support. **CHL**: Conceptualization, Formal analysis, Data curation, Resources, Funding acquisition, Writing – review & editing.

## Conflicts of Interest

The authors declare no conflicts of interest.

## Supporting information




**Supporting File**: advs77758‐sup‐0001‐SuppMat.pdf.

## Data Availability

The raw and processed data of scRNA‐seq analysis are available at the GEO repository (GSE316376).
