# Supplementary material for: Multi‐Tissue Crosstalk Regulating Meniscus Healing Under Inflammation and Physiological Loading
Source: Adv Sci (Weinh). 2026 Sep 16:e77758. Online ahead of print. doi: 10.1002/advs.77758 (PMC13583097; doi:10.1002/advs.77758)
Supplement: Supplementary file 1 [file ADVS-9999-e77758-s001.pdf]

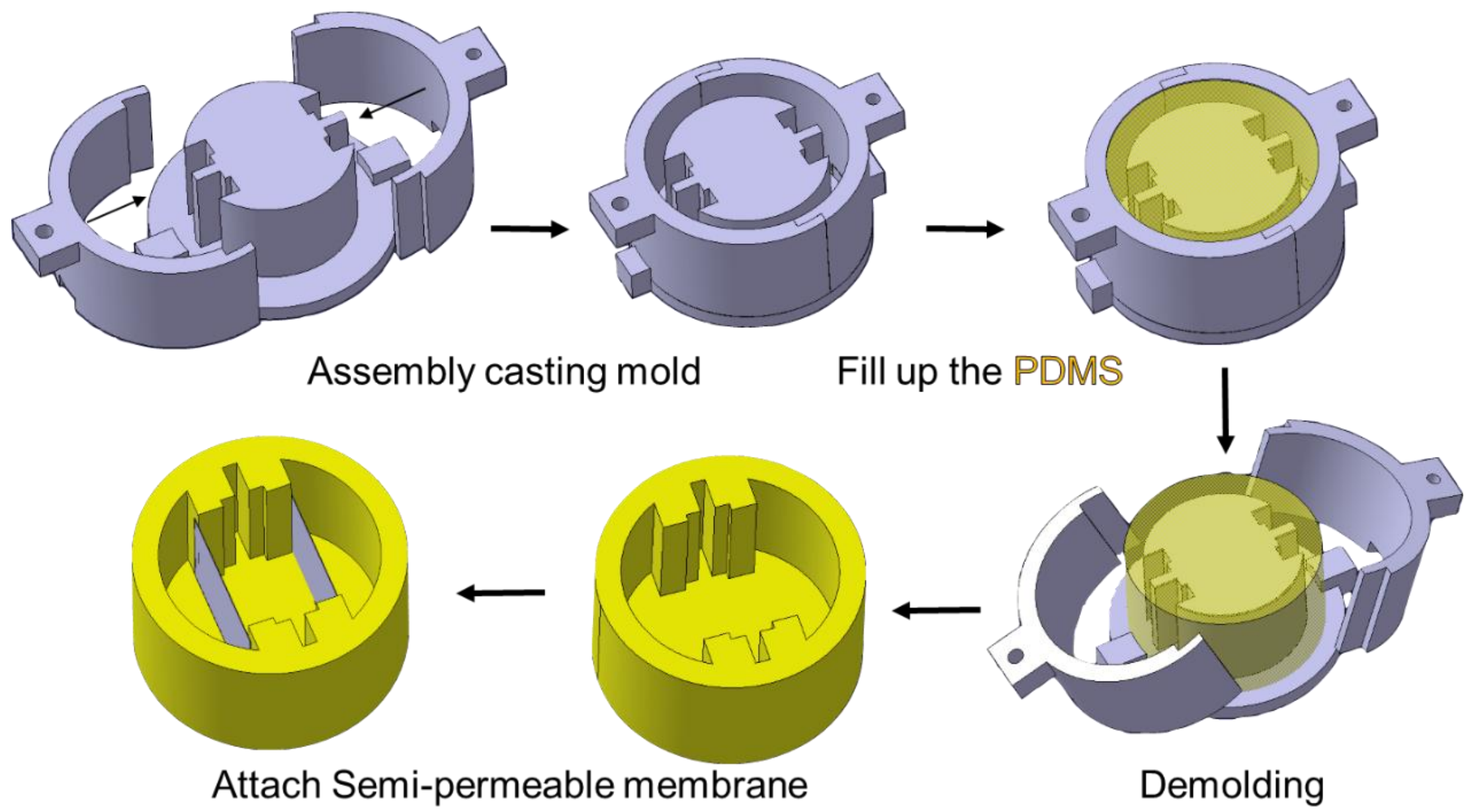

Supplementary Figure 1. Fabrication of MoC with multi-tissue compartments.

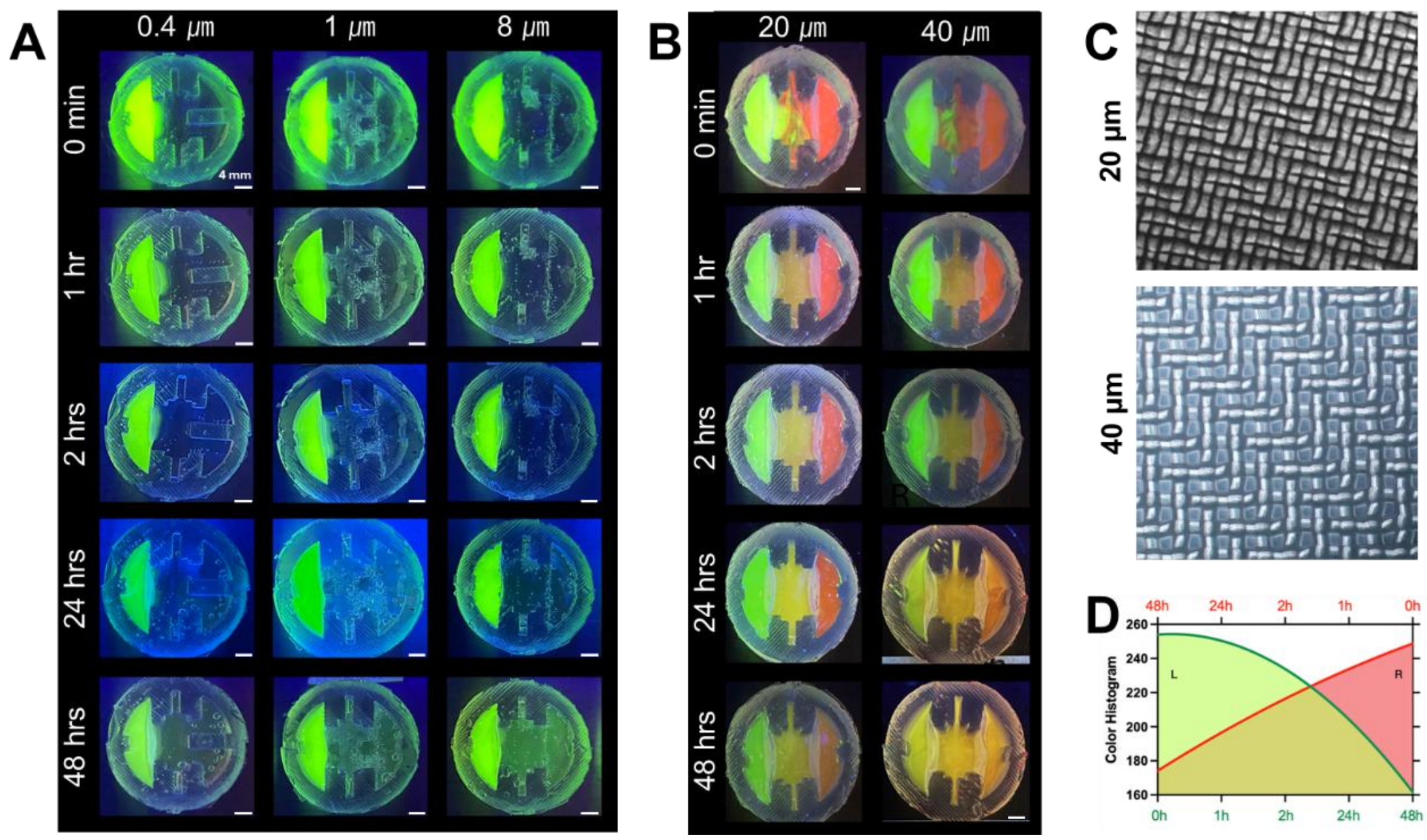

Supplementary Figure 2. Permeability test of semi-permeable nylon membranes with different porosity. Fluorescent-labeled dextran (70 kDa) was applied to evaluate its diffusion cross the tissue compartments. Green dextran was applied on the left tissue compartment separated by smaller pores (**A**). Red and green dextrans were applied on the right and left tissue compartments, respectively (**B**). Microscopic images 20 μm and 40 μm porous membranes (**C**). Color histogram over time with 20 μm membrane (**D**).

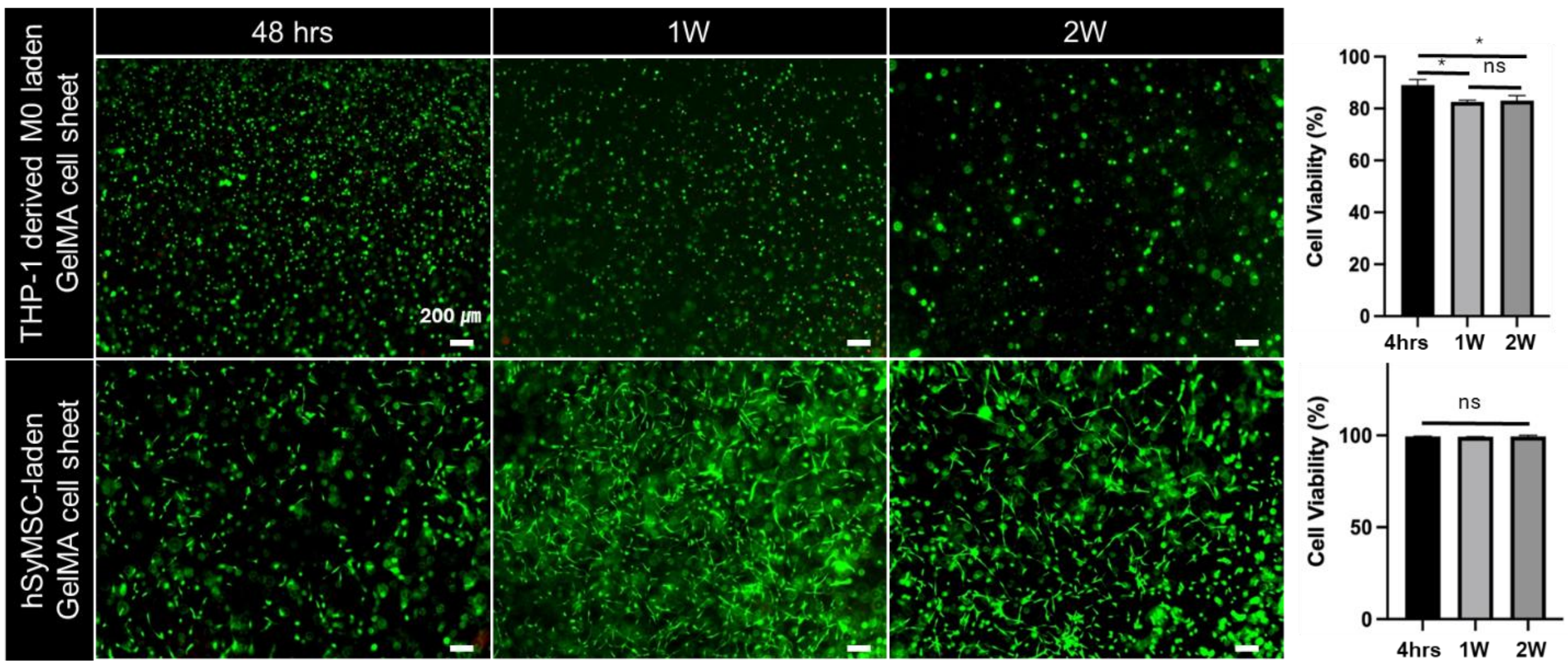

Supplementary Figure 3. Viability of syMSCs and THP-1 derived macrophages in bioprinted syM by 2 weeks (Live/dead assay).

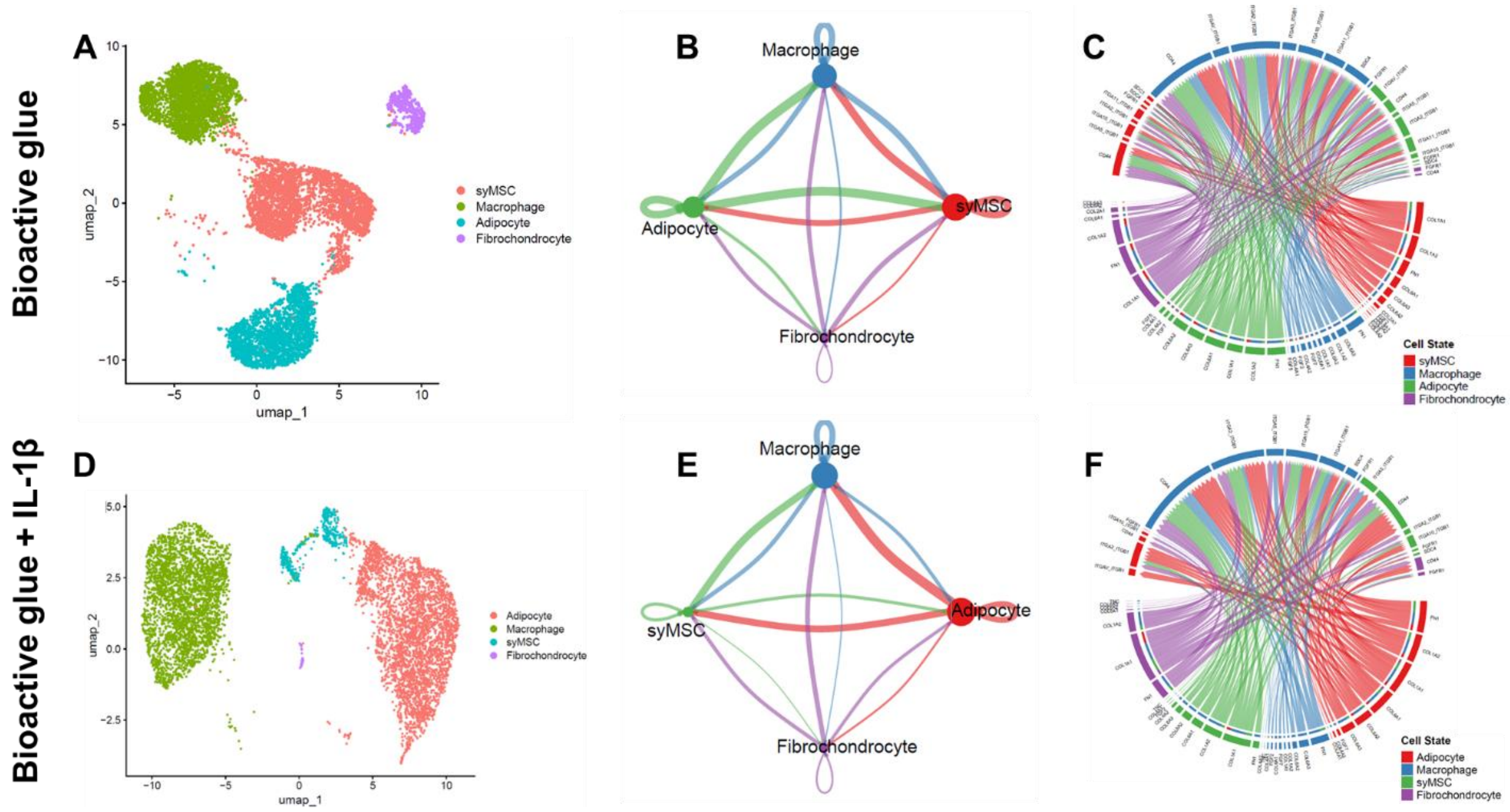

Supplementary Figure 4. The scRNA-seq CellChat analysis for MoC with meniscus injury treated by bioactive glue with and without IL-1β. UMAPs (**A, D**) show identified cell clusters, Circle plots show the intensity of cell-cell interactions (**B, E**), and Chord plots show signaling pathways involved in the cell-cell communications (**C, F**).

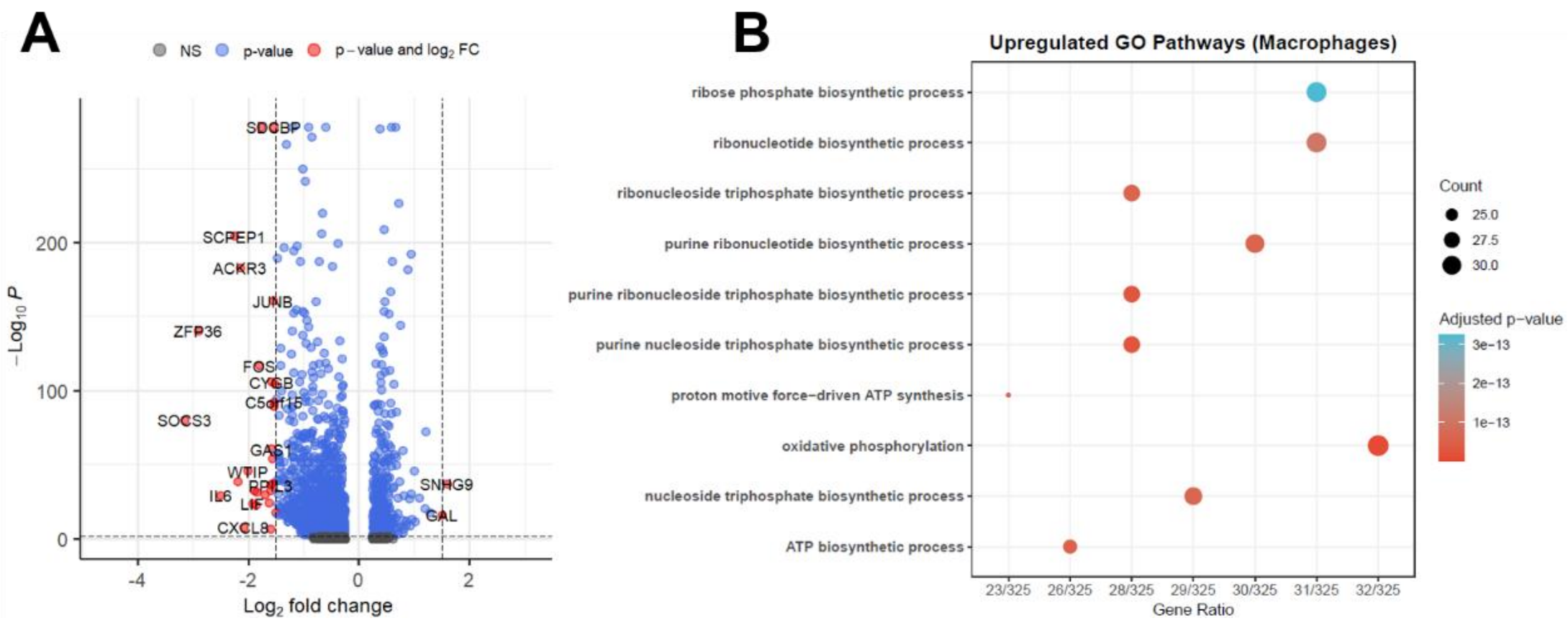

Supplementary Figure 5. DEG and GO enrichment in macrophages with and without IL-1β.

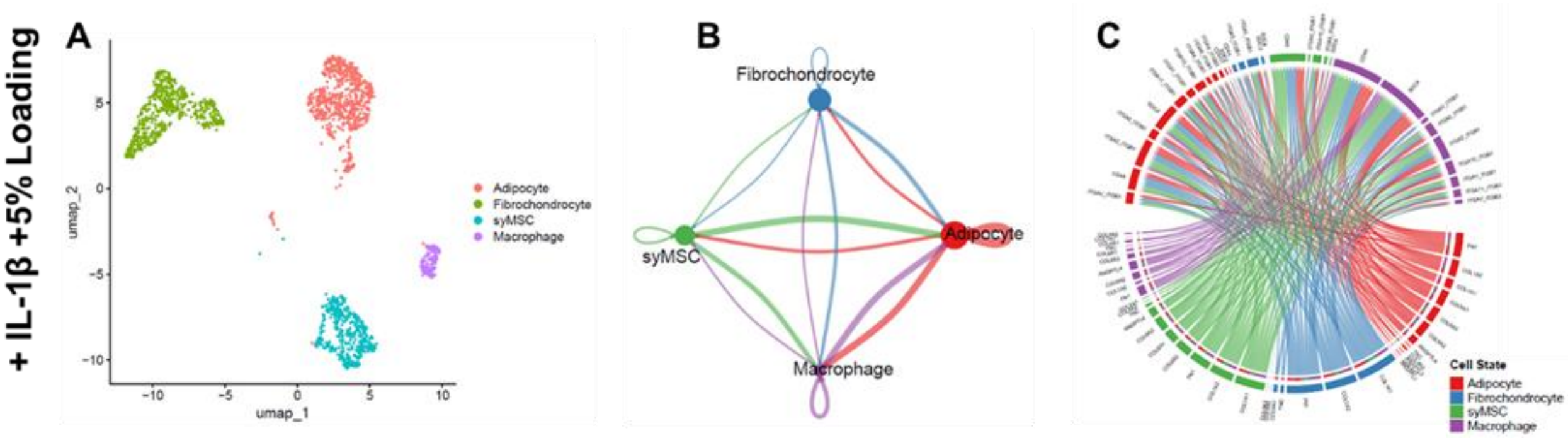

Supplementary Figure 6. scRNA-seq and CellChat analysis for MoC with IL-1β and 5% physiological loading: UMAP (**A**), Circle plot of cell-cell interactions (**B**), and Chord plot of cell-cell communication signals (**C**).

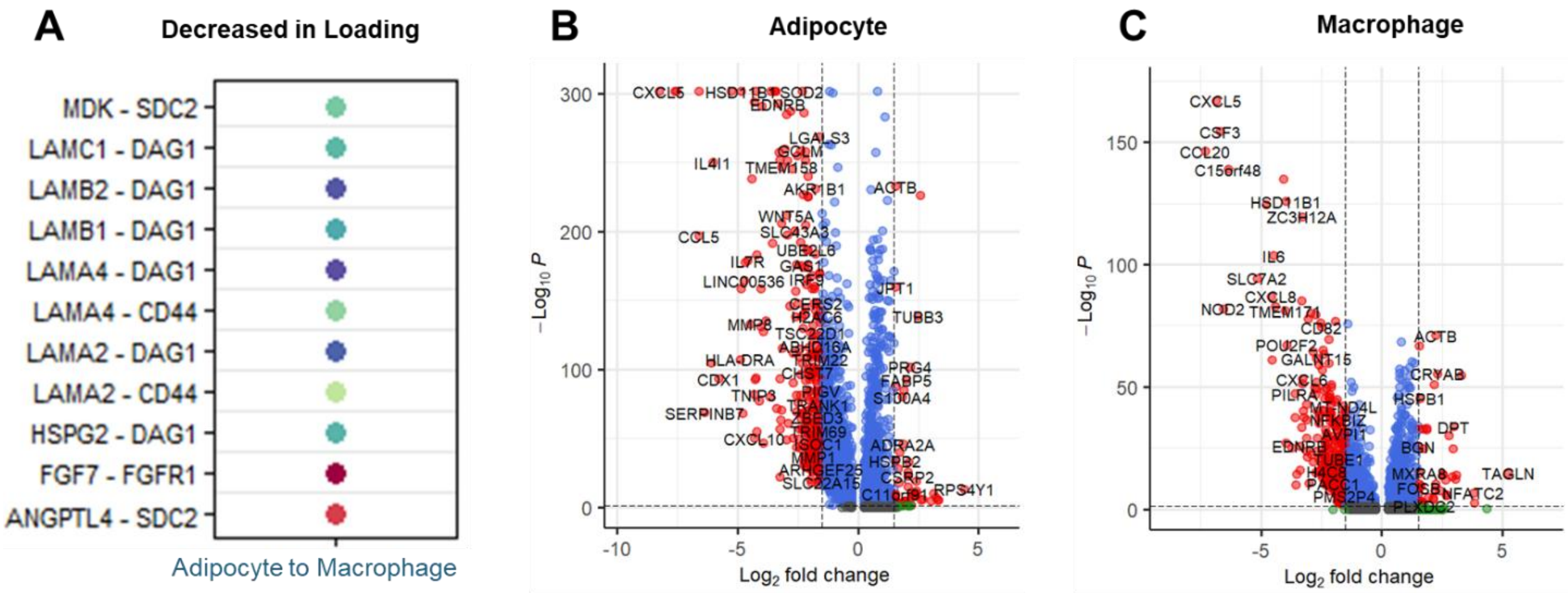

Supplementary Figure 7. Bubble plot shows decreased ligand-receptor pairs in adipocytes by mechanical loading (**A**). Volcano plots show DEGs in adipocytes (**B**) and macrophages (**C**). Threshold lines in volcano plots: $|\log_2$ fold change$| > 1.5$ and adjusted $p < 0.05$.

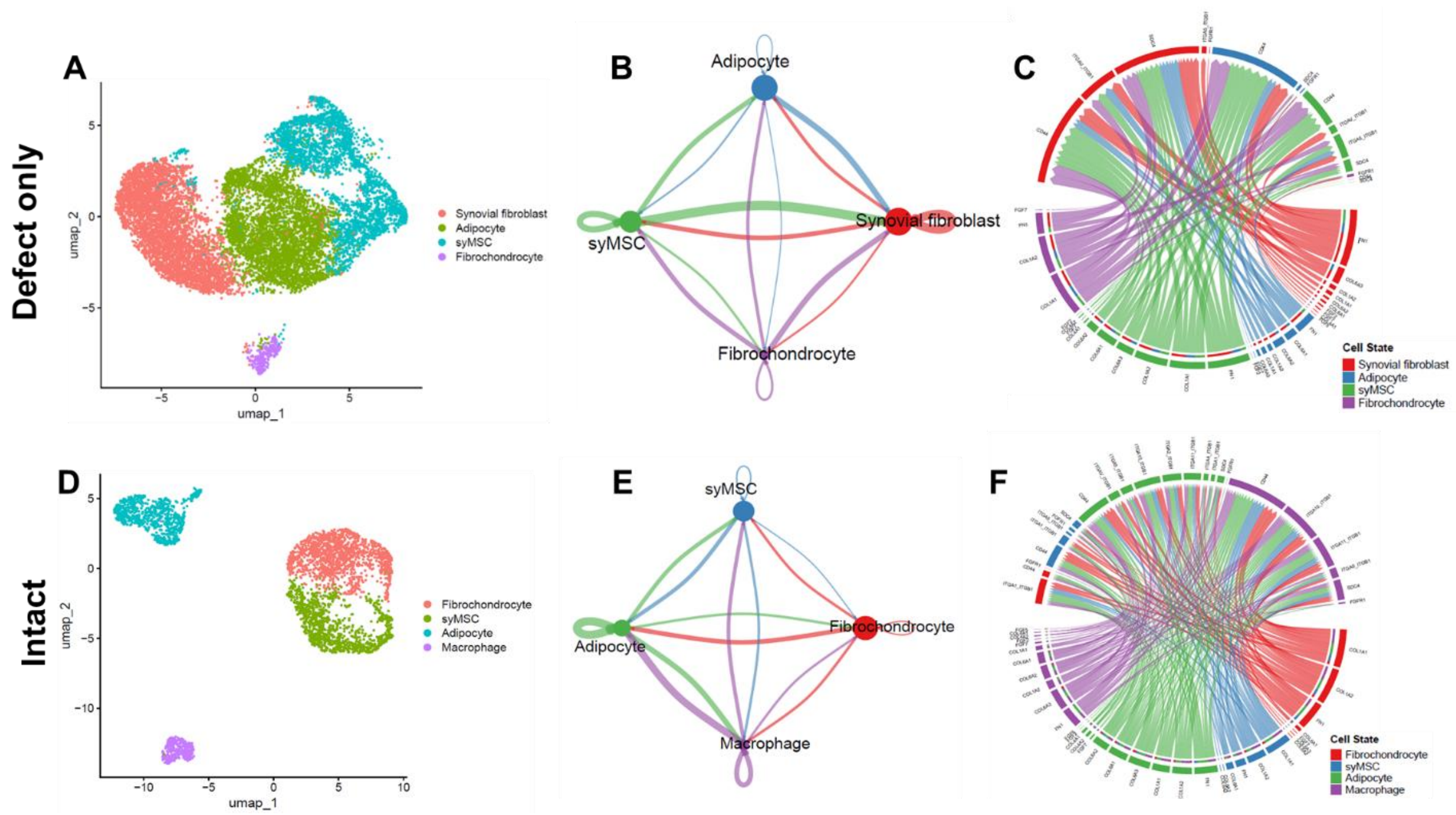

Supplementary Figure 8. scRNA-seq and CellChat analysis for defect only and intact group: UMAPs (**A, D**), Circle plot for cell-cell interactions (**B, E**), and Chord plots for cell-cell communication signals (**C, F**).

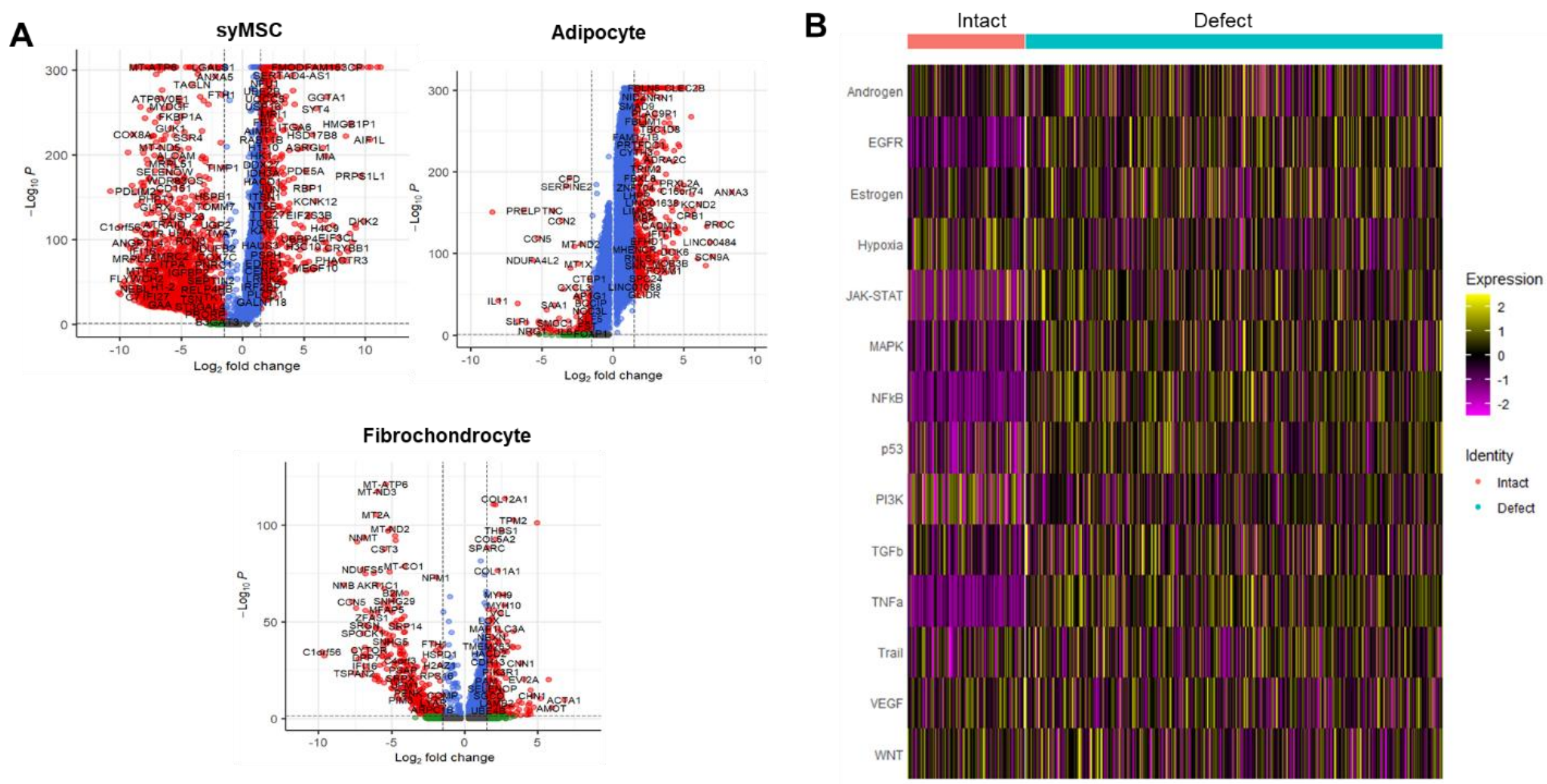

Supplementary Figure 9. DEGs in syMSC, adipocyte, and fibrochondrocyte between the defect only and intact (**A**). PROGENy analysis for 14 core pathways, focused on top 500 downstream target genes (**B**). Threshold lines in volcano plots: $|\log_2$ fold change$| > 1.5$ and adjusted $p < 0.05$.

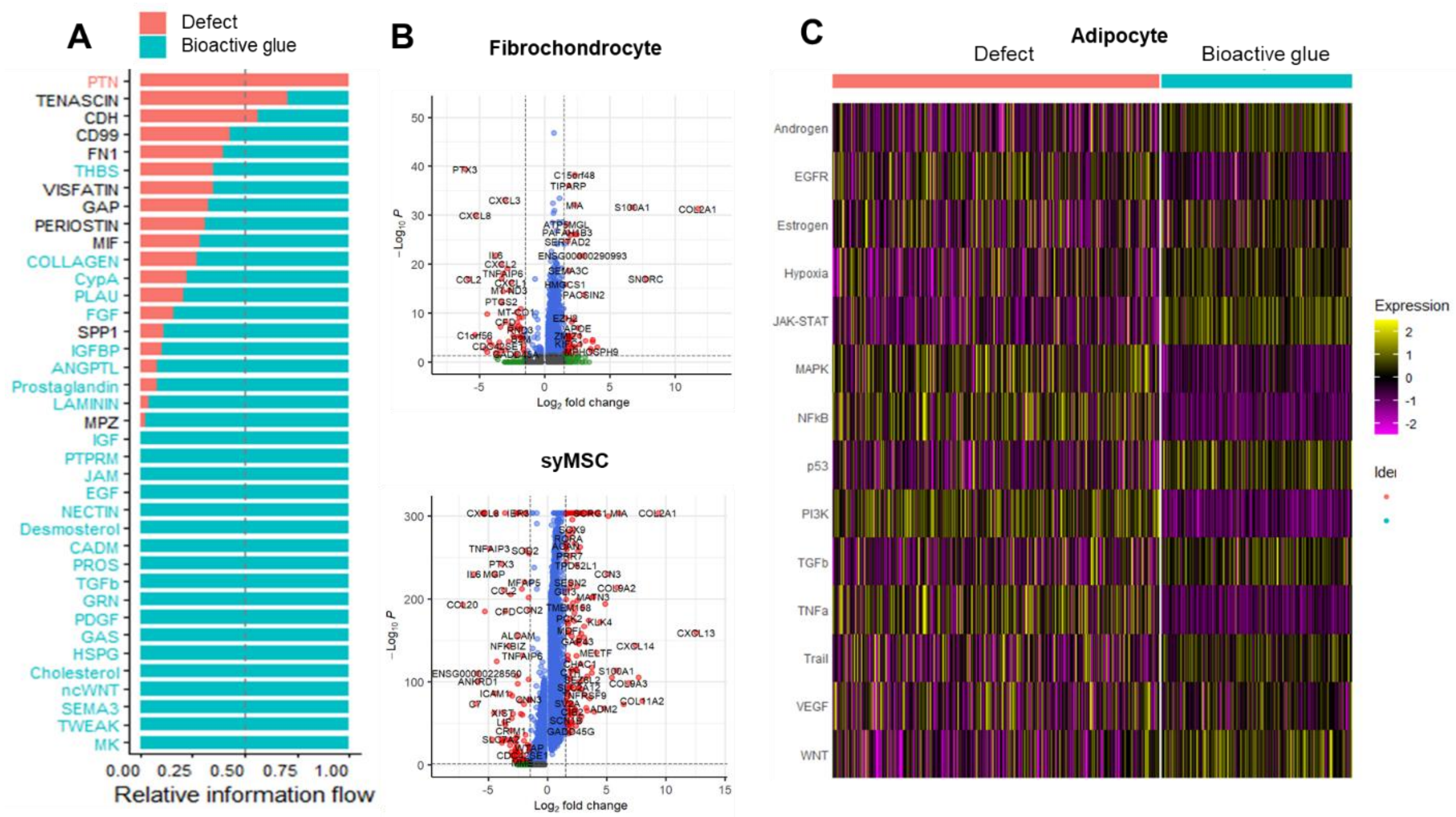

Supplementary Figure 10. Comparative analysis between bioactive glue treatment and defect alone in MoC: Relative cell-cell interaction signal flow (**A**), DEGs (**B**), and PROGENy for adipocytes (**C**). Threshold lines in volcano plots: $|\log_2$ fold change$| > 1.5$ and adjusted $p < 0.05$.

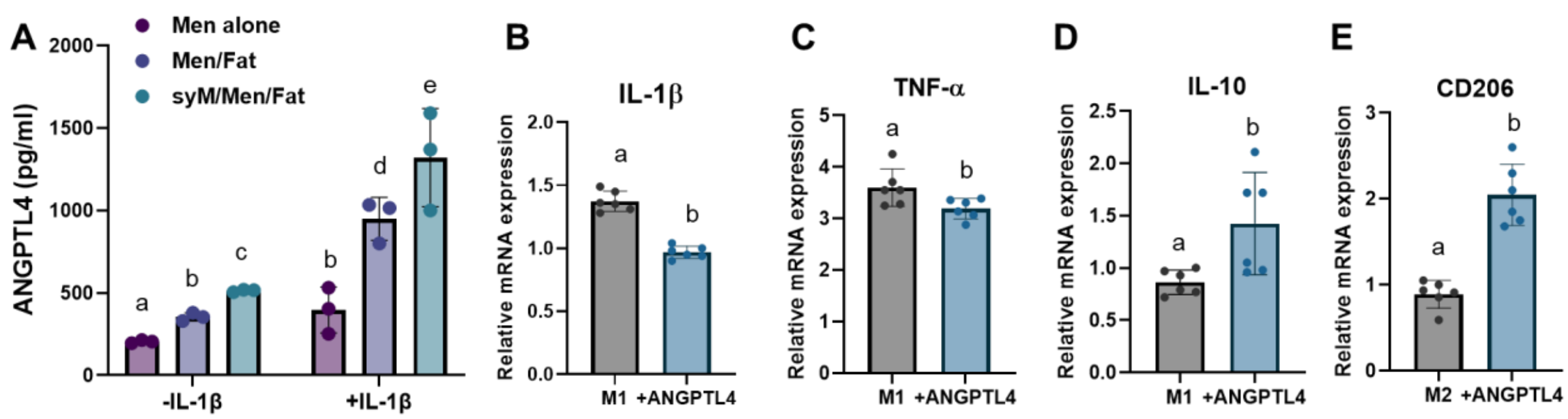

Supplementary Figure 11. Validation for the anti-inflammatory roles of ANGPTL-4. The concentration of ANGPTL-4 is significantly increased in three-tissue co-culture group with IL-1β (**A**) (n = 3 per group). Application of ANGPTL-4 (200 ng/ml) significantly reduced M1 markers (**B, C**) and increased M2 marker, IL-10 (**D**) and CD206 (**E**) (n = 6 per group). Different letters indicate significant differences ($p<0.05$).

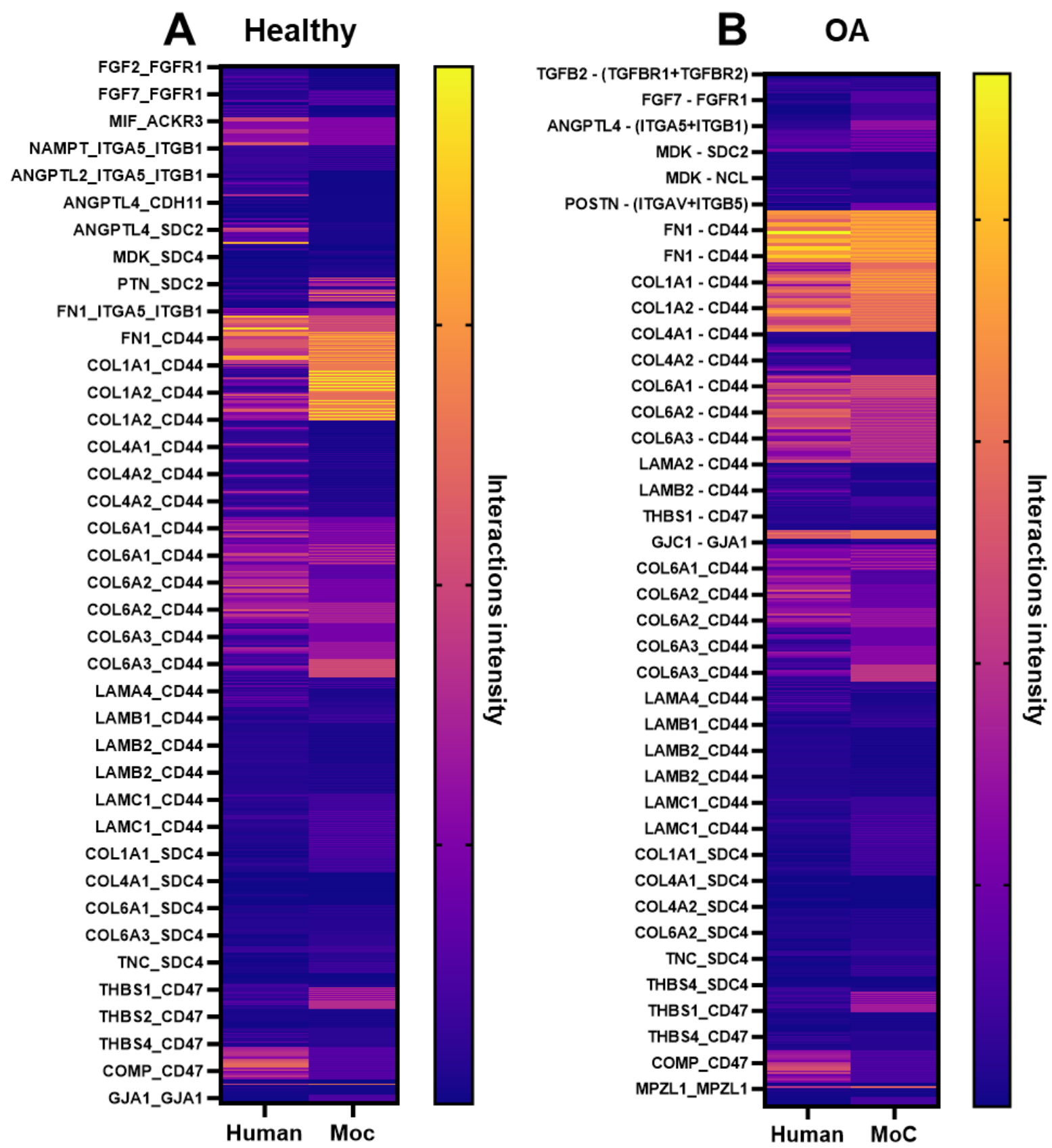

Supplementary Figure 12. Matched cell-cell interactions (ligand-receptor pairs) in cell source/target-matched dataset: (A) Healthy human vs. intact MoC (1,032 interactions), (B) OA human vs. MoC with meniscus injury and IL-1β (470 interactions). Labels do not show entire interactions due to display restriction.
